# Transcriptomic analysis of the human habenula in schizophrenia

**DOI:** 10.1101/2024.02.26.582081

**Published:** 2024-02-27

**Authors:** Ege A. Yalcinbas, Bukola Ajanaku, Erik D. Nelson, Renee Garcia-Flores, Kelsey D. Montgomery, Joshua M. Stolz, Joshua Wu, Heena R. Divecha, Atharv Chandra, Rahul A. Bharadwaj, Svitlana Bach, Anandita Rajpurohit, Ran Tao, Joo-Heon Shin, Joel E. Kleinman, Thomas M. Hyde, Daniel R. Weinberger, Louise A. Huuki-Myers, Leonardo Collado-Torres, Kristen R. Maynard

**Affiliations:** 1. Lieber Institute for Brain Development, Johns Hopkins Medical Campus, Baltimore, MD, 21205, USA; 2. Department of Psychiatry and Behavioral Sciences, Johns Hopkins School of Medicine, Baltimore, MD, 21205, USA; 3. Department of Neurology, Johns Hopkins School of Medicine, Baltimore, MD, 21205, USA; 4. The Solomon H. Snyder Department of Neuroscience, Johns Hopkins School of Medicine, Baltimore, MD, 21205, USA; 5. McKusick-Nathans Department of Genetic Medicine, Johns Hopkins School of Medicine, Baltimore, MD, 21205, USA; 6. Department of Biostatistics, Johns Hopkins Bloomberg School of Public Health, Baltimore, MD, 21205, USA; 7. Center for Computational Biology, Johns Hopkins University, Baltimore, MD, 21205, USA

## Abstract

**Importance:**

Habenula (Hb) pathophysiology is involved in many neuropsychiatric disorders, including schizophrenia. Deep brain stimulation and pharmacological targeting of the Hb are emerging as promising therapeutic treatments. However, little is known about the cell type-specific transcriptomic organization of the human Hb or how it is altered in schizophrenia.

**Objective:**

To define the molecular neuroanatomy of the human habenula and identify transcriptomic changes in individuals with schizophrenia compared to neurotypical controls.

**Design, Setting, and Participants:**

This study utilized Hb-enriched postmortem human brain tissue. Single nucleus RNA-sequencing (snRNA-seq) and single molecule fluorescent *in situ* hybridization (smFISH) experiments were conducted to identify molecularly defined Hb cell types and map their spatial location (n=3–7 donors). Bulk RNA-sequencing and cell type deconvolution were used to investigate transcriptomic changes in Hb-enriched tissue from 35 individuals with schizophrenia and 33 neurotypical controls. Gene expression changes associated with schizophrenia in the Hb were compared to those previously identified in the dorsolateral prefrontal cortex (DLPFC), hippocampus, and caudate.

**Main Outcomes and Measures:**

Semi-supervised snRNA-seq cell type clustering. Transcript visualization and quantification of smFISH probes. Bulk RNA-seq cell type deconvolution using reference snRNA-seq data. Schizophrenia-associated gene differential expression analysis adjusting for Hb and thalamus fractions, RNA degradation-associated quality surrogate variables, and other covariates. Cross-brain region schizophrenia-associated gene expression comparison.

**Results:**

snRNA-seq identified 17 cell type clusters across 16,437 nuclei, including 3 medial and 7 lateral Hb populations. Cell types were conserved with those identified in a rodent model. smFISH for cell type marker genes validated snRNA-seq Hb cell types and depicted the spatial organization of subpopulations. Bulk RNA-seq analyses yielded 45 schizophrenia-associated differentially expressed genes (FDR < 0.05), with 32 (71%) unique to Hb-enriched tissue.

**Conclusions:**

These results identify topographically organized cell types with distinct molecular signatures in the human Hb. They further demonstrate unique transcriptomic changes in the epithalamus associated with schizophrenia, thereby providing molecular insights into the role of Hb in neuropsychiatric disorders.

## Introduction

The habenula (Hb) is a small epithalamic brain structure implicated in several neuropsychiatric conditions, including mood and anxiety disorders, substance use disorders, and schizophrenia (SCZD) ^1–3^. Anatomically, it is divided into molecularly and functionally distinct medial (MHb) and lateral (LHb) subdivisions with discrete connections to monoaminergic hubs, including dopamine, serotonin, and norepinephrine centers ^2^. As a circuit hub involved in motivated behaviors and affective states, the Hb has emerged as a promising therapeutic target ^4–9^. Imaging studies in SCZD patients have sought to identify aberrations in Hb anatomy, connectivity, and activity, but challenges in segmenting this small midline structure have hindered investigation into its subregions and limited conclusive findings ^10–14^. Similarly, there is a paucity of molecular studies in postmortem human Hb due to its challenging size and location ^15–18^. While bulk RNA-sequencing (RNA-seq) studies identified gene expression changes associated with SCZD in other brain regions ^19–21^, the molecular landscape of the Hb in SCZD has not yet been investigated.

Single cell RNA-sequencing studies in animal models found diverse cell populations with unique molecular signatures across LHb and MHb ^22–24^. However, it is unclear whether these Hb cell types are conserved in the human brain. This information is important for understanding biological vulnerabilities underlying human disease, which is critical for improving translational research outcomes ^25^. Given the central role of the Hb in modulating dopaminergic, serotonergic, and cholinergic pathways, molecular profiling in this region can reveal novel mechanistic insights into disease pathogenesis, especially regarding the nuanced role of dopamine dysregulation in SCZD. For example, the fact that many antipsychotic medications act through dopamine and serotonin receptors warrants a deeper exploration of Hb cell types and signaling pathways contributing to downstream monoaminergic dysfunction^26^. Additionally, SCZD is associated with nicotine dependence, and smoking is reported to improve cognitive symptoms ^27–29^. The MHb is enriched in cholinergic populations implicated in nicotine addiction ^30–32^, but a link between cholinergic signaling, nicotine dependence, and psychotic disorders as it relates to the human Hb has yet to be elucidated ^33–36^.

To address these gaps in knowledge, we generated the first single cell transcriptomic atlas of the healthy human Hb and evaluated the topography and cross-species convergence of MHb and LHb cell types. To understand molecular changes in the human Hb associated with SCZD, we performed bulk RNA-seq in Hb-enriched tissue from SCZD and neurotypical control donors. We identified gene expression changes unique to Hb-enriched tissue compared to other brain regions in the context of SCZD. Our findings support previous hypotheses of SCZD etiology that implicate neurodevelopmental processes ^37–40^, while providing additional molecular insights into Hb cell types that may contribute to SCZD pathogenesis.

## Methods

### Postmortem human brain tissue

Postmortem human brain tissue was collected from the Lieber Institute for Brain Development (LIBD) Human Brain and Tissue Repository through the following locations and protocols at the time of autopsy. Thirty-one samples were collected at LIBD between May 2013 and December 2017, twenty-eight of which were consented through the Office of the Chief Medical Examiner of the State of Maryland, under the Maryland Department of Health’s IRB protocol #12–24. The remaining three samples were consented through the Department of Pathology at Western Michigan University Homer Stryker MD School of Medicine, under WCG IRB protocol #20111080. Thirty-seven additional samples were consented through the National Institute of Mental Health Intramural Research Program (NIH protocol #90-M-0142), and were acquired by LIBD via material transfer agreement. Demographics and other information for the 69 donors are listed in ETable 1. Details of tissue acquisition, handling, processing, dissection, clinical characterization, diagnoses, neuropathological examinations, and quality control measures are previously described^41^. Fresh frozen Hb-enriched tissue samples were microdissected, pulverized, and stored at −80°C prior to snRNA-seq and bulk RNA-seq experiments. For single molecule fluorescent *in situ* hybridization (smFISH) experiments, larger tissue blocks were removed and kept intact for cryosectioning.

### Single nucleus RNA-sequencing (snRNA-seq) and analysis

**Figure 1:**
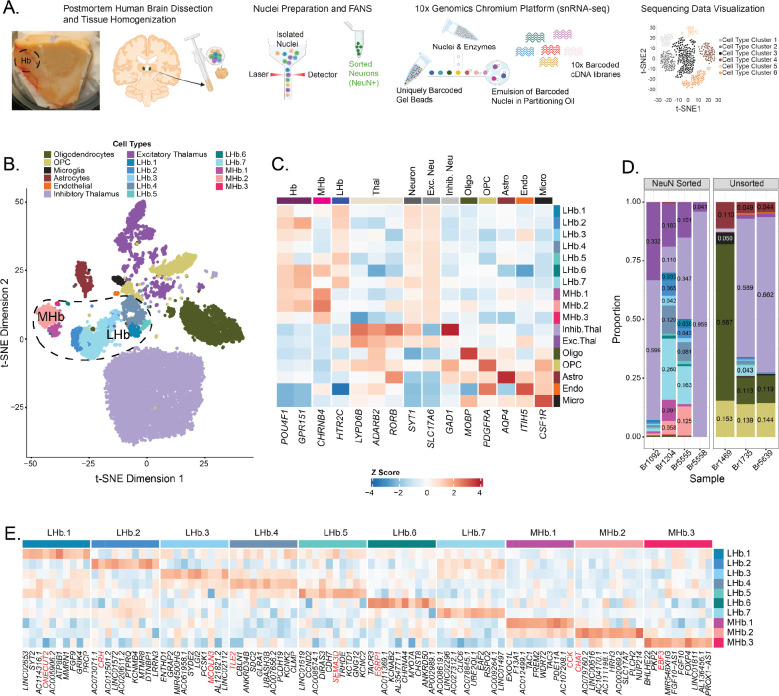
Single cell transcriptomic atlas of the human habenula (Hb). **A)** Overview of nuclei preparation and single nucleus RNA-sequencing (snRNA-seq) data generation using the 10x Genomics Chromium Single Cell Gene Expression platform **B)** t-SNE plot of 16,437 quality-controlled single nucleus transcriptomes (colored by cell type) from the epithalamus of 7 neurotypical control donors. Dashed circle highlights lateral (L) and medial (M) Hb cell type clusters (n=2,214 LHb and n=710 MHb nuclei). **C)** Heatmap depicting expression of known marker genes (x-axis) that were used to annotate broad cell types as well as habenula vs. thalamic neurons. The color of each square (blue to red) indicates z-scored relative expression of the corresponding marker gene. **D)** Proportion plot displaying cell type composition of each sample. NeuN+ sorted samples were enriched for neurons and therefore contain more neuronal cell types. **E)** Heatmap highlighting the top 10 mean ratio marker genes (x-axis) for each identified LHb and MHb neuronal subpopulation. The color of each square (blue to red) indicates z-scored relative expression of the corresponding marker gene. Marker genes highlighted in red text were further validated using single molecule fluorescent *in situ* hybridization (smFISH, see Fig 3).

Nuclei isolation was performed on seven neurotypical control samples also included in the bulk RNA-seq study (ETable 1) as previously described ^42^. Nuclei were stained with propidium iodide (Invitrogen, Thermo Fisher) and sorted using fluorescence-activated nuclei sorting (FANS). Four samples were also stained with anti-NeuN (Millipore Sigma) for enrichment of neuronal nuclei. Using a BioRad S3e cell sorter, ~9K nuclei were sorted per sample and loaded onto individual lanes of the 10x Genomics Chromium instrument. GEMs were produced, cDNA was generated, and libraries were prepared according to manufacturer’s instructions and sequenced on an Illumina Nextseq (ETable 2). Reads were aligned with *CellRanger* (10x Genomics), empty droplets were identified with *DropletUtils*^43^, and nuclei quality was assessed by examining library size, number of detected genes, doublet scores, and fraction of reads mapping to mitochondrial and ribosomal genes (Supplementary Figure 1–2). Batch correction was performed with *Harmony* ^44^, and dimension reduction with a generalized linear model for principal component analysis ^45^ (Supplementary Figure 3–5). Clustering was performed with graph-based clustering from *scran* ^46^. Fine-resolution Hb clusters were maintained, but other cell types were collapsed into broader categories yielding 17 annotated cell type clusters containing 16,437 nuclei (Fig 1B). Marker genes for each Hb subcluster were selected with the *Mean Ratio* method (Fig 1E; ETable 3).

### Single molecule fluorescent *in situ* hybridization (smFISH)

smFISH experiments were performed on postmortem human brain tissue using RNAScope Multiplex Fluorescent Reagent Kit v2 (Advanced Cell Diagnostics) and 4-Plex Ancillary Kit as previously described ^47^. Briefly, Hb tissue from three independent donors (ETable 4) was cryosectioned for four experiments targeting different Hb cell type clusters identified using our snRNA-seq dataset (Fig 1, Supplementary Figure 6A, Supplementary Figure 7A, Supplementary Figure 8A, ETable 5). Two probe combinations targeted LHb subclusters and two probe combinations targeted MHb subclusters (see Supplemental Methods). Briefly, tissue sections (n=2–4 technical replicates per donor) were fixed in 10% Normal Buffered Formalin (NBF) solution, rinsed in phosphate buffered saline (PBS), and dehydrated in a series of ethanol dilutions. Sections were treated with hydrogen peroxide, rinsed in PBS, and permeabilized with Protease IV. Following PBS wash steps, sections were hybridized with RNAScope probes, and signal amplification was performed according to manufacturer’s instructions. A distinct Opal fluorophore (Perkin Elmer, 1:500) was assigned to each probe and sections were counterstained with DAPI (4′,6-diamidino-2-phenylindole) to label nuclei. For each tissue section, a 20X max-intensity projected z-stack image was obtained with a Nikon AXR confocal microscope using spectral imaging and linear unmixing as previously described ^47^. Confocal imaging data were saved as .nd2 files for downstream quantitative analysis using HALO software (Indica labs). The distributions of estimated marker gene expression (“copies”) across cell objects were used to quantify abundant expression (top ranks) and delineate thresholds for data display (Supplementary Figure 9).

### Bulk RNA-sequencing

Total RNA was extracted from samples using the Qiagen AllPrep DNA/RNA/miRNA Universal Kit (Cat No./ID: 80224). Paired-end strand-specific sequencing libraries were prepared for 34 Control and 35 SCZD samples from 300 ng total RNA using the TruSeq Stranded Total RNA Library Preparation kit with Ribo-Zero Gold ribosomal RNA depletion. For quality control, synthetic External RNA Controls Consortium (ERCC) RNA Mix 1 (Thermo Fisher Scientific) was spiked into each sample. Libraries were sequenced on an Illumina HiSeq 3000 producing 30.14 to 645.8 million (median 90.65, mean 150.07) 100-bp paired-end reads per sample. Bulk RNA-seq FASTQ files were aligned to Gencode v25 ^48^ using *SPEAQeasy* ^49^, a *Nextflow* ^50^ workflow for *HISAT2* RNA-seq alignments ^51^. All samples passed quality control based on sequencing metrics (Supplementary Figure 10).

### Deconvolution

Given the small size of the Hb and inclusion of surrounding thalamic tissue during microdissection, cell type deconvolution of the bulk RNA-seq data was performed with *Bisque* ^52^. Medial and lateral Hb subclusters were collapsed and the top 25 mean ratio marker genes for each cell type were selected for deconvolution (ETable 6). For validation purposes, we also calculated the standard log2 Fold Change for selected marker genes ^46^ with a model adjusting for the donor ID and contrasting a given cell type against all the rest (Supplementary Figure 11). One bulk RNA-seq sample (Br5572) was excluded from further analyses as it was predicted to consist entirely of thalamic tissue (Supplementary Figure 12).

### Cross-brain region bulk RNA-seq data integration

To examine the unique gene expression profile of the Hb, the Hb-enriched thalamic bulk RNA-seq samples were compared to bulk RNA-seq samples from 11 other brain regions: Amygdala ^53^, basolateral amygdala (BLA), cornu ammonis (CA), caudate ^20^, dorsal anterior cingulate cortex (dACC), dentate gyrus (DG) ^19^, dorsolateral prefrontal cortex (DLPFC) ^21^, hippocampus (HIPPO) ^21^, medial amygdala (MeA), medial prefrontal cortex (mPFC), and subgenual anterior cingulate cortex (sACC) ^53^. These LIBD samples came from donors with the same demographics: male, neurotypical, adult at age of death (17–70 years), and of European descent (EUR/CAUC). Principal components were computed with log normalized gene expression (Fig 4B-C).

### Bulk RNA-seq Differential Gene Expression (DGE)

Gene level differential expression analysis between SCZD and Control bulk RNA-seq samples was performed using the limma-voom method ^54^ (Fig 4D) while adjusting for demographics, quality metrics, and quality surrogate variables ^55,56^ To assess the variability in SCZD-associated DEGs across brain regions the Hb (+ Thal.) DGE results were compared to statistics from previous SCZD bulk RNA-seq studies: DLPFC, HIPPO ^21^, DG ^19^, and caudate ^20^ (ETable 7, Fig 4E). Bulk and snRNA-seq gene expression data can be interactively explored through *iSEE*-powered websites https://github.com/LieberInstitute/Habenula_Pilot#interactive-websites ^57^.

## Results

### IDENTIFICATION OF DISTINCT HUMAN HB CELL TYPES

To generate a molecular atlas of human Hb cell populations, we performed snRNA-seq on neuronal (NeuN+) and non-neuronal nuclei from Hb-enriched tissue from 7 neurotypical control donors (Fig 1A; ETable 1). Following quality control, 16,437 single nucleus transcriptomes were included in downstream analyses and annotated at broad resolution based on canonical marker gene expression (Supplementary Figure 1–5; ETable 2). Graph-based clustering identified 17 molecularly-defined cell types, including non-neuronal populations (oligodendrocytes, astrocytes, microglia, oligodendrocyte precursor cells, and endothelial cells; n=4,101 nuclei), thalamic neurons (excitatory and inhibitory; n=9,412 nuclei), and habenula neurons (7 lateral [L] and 3 medial [M] Hb subpopulations; n=2,924 nuclei) (Fig 1B,C; Supplementary Figure 13–13; Supplemental Methods). Principal component analysis revealed that the second and third components of variation differentiated Hb from thalamic neurons, and neuronal from non-neuronal cell types, respectively (Supplementary Figure 15). The small size and midline location of the Hb resulted in variability in epithalamus dissections across donors; thus, our samples contained varying proportions of thalamic and Hb neurons, as well as neuronal versus non-neuronal cell types (Fig 1D). However, neuronal enrichment with NeuN+ sorting in a subset of samples increased the capture rate of Hb neurons, and we observed the presence of LHb and medial MHb neuronal subpopulations in most samples.

Broad-level cell labels were assigned based on previously established marker genes from human, rodent, and zebrafish studies (Fig 1C) ^23,58–61^. As expected, *POU4F1* and *GPR151* were enriched in human Hb subpopulations compared to thalamic and non-neuronal populations ^17,62–65^. We also identified *CHRNB4* and *HTR2C* as marker genes for MHb and LHb subpopulations, respectively (ETable 6). *CHRNB4* encodes a nicotinic acetylcholine receptor (nAChR) subunit, and is an MHb marker gene in both rodents and humans along with *CHRNA3*, another nAChR subunit listed as a top mean ratio marker for MHb ^23^. These two genes are in a conserved gene locus implicated in nicotine dependence ^66,67^. *HTR2C,* which encodes a G-protein-coupled receptor (GPCR) serotonin receptor, is also a rodent LHb marker gene ^22,23^. To better understand cell type diversity within anatomical subdivisions of Hb, we identified top mean ratio marker genes for the 10 LHb and MHb subpopulations (Fig 1E; ETable 3; Supplemental Methods). This allowed us to gain functional insights into different LHb and MHb subpopulations, such as the identification of cholinergic neurons (MHb.2) expressing high levels of *CHAT* and the identification of a LHb.2 subpopulation expressing high levels of *CRH* and *OPRM1*.

### CONSERVATION OF HB CELL TYPES FROM PRECLINICAL RODENT MODEL IN HUMAN HB

**Figure 2:**
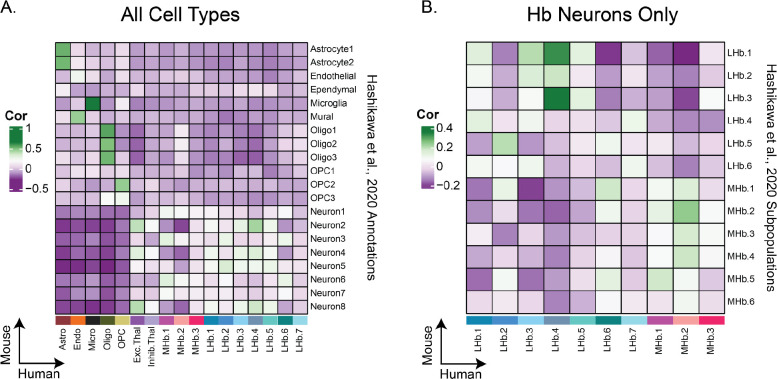
Conservation of LHb and MHb neuronal subpopulations from preclinical rodent model in human Hb. **A)** Pearson correlation heatmap of *t*-statistics obtained from a cell type differential gene expression (DGE) analysis conducted on a mouse Hb scRNA-seq dataset ^22^ along the y-axis, and *t*-statistics from a DGE analysis of our human cell types identified in Fig 1B along the x-axis. Green squares show positive correlation, i.e. convergence of cell type-specific gene expression patterns between species. **B)** Correlation heatmap obtained similarly to A, but subset to Hb neuronal subpopulations.

To evaluate whether Hb cell types found in rodents are conserved in human Hb, we conducted a cross-species correlation analysis with mouse Hb single cell RNA-seq data ^22^. We observed positive correlations between the human and mouse datasets across broad cell type categories, including astrocytes, microglia, oligodendrocytes, and neurons (Fig 2A). A correlation analysis restricted to human Hb (3 MHb, 7 LHb) and mouse Hb neuronal cell types (6 MHb, 6 LHb) revealed some cross-species convergence of Hb neurons (Fig 2B). Human MHb subpopulations were most strongly correlated with mouse MHb subpopulations, and the same relationship held true for the LHb subpopulations, with the exception of human LHb.6 which most strongly correlated with mouse MHb.1. Of particular interest was the convergence of human MHb.1 and MHb.2 with mouse MHb subpopulations. Human MHb.1 neurons express relatively high levels of *CCK*, *TAC1*, and *TAC3,* which encode various neuropeptides; human MHb.2 neurons are cholinergic as evidenced by relative high levels of *CHAT* expression (Fig 1E). Neuronal subpopulations enriched in these marker genes were previously identified in rodent Hb ^22,23^. Among the LHb subpopulations, human LHb.4, LHb.3, and LHb.2 had the strongest positive correlation with mouse LHb.3, LHb.1, and LHb.5, respectively, suggesting cross-species convergence of these molecularly distinct neuronal cell types.

### TOPOGRAPHY OF HUMAN HB CELL TYPES

**Figure 3:**
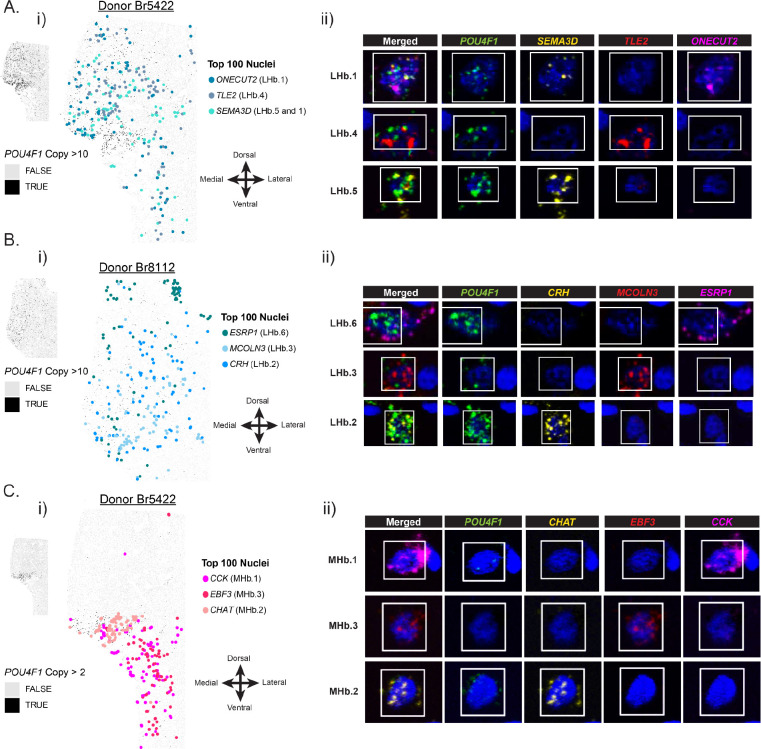
Spatial organization of molecularly-defined LHb and MHb neuronal subpopulations in postmortem human tissue. Spatial plots displaying the topographic arrangement of top cells that express the marker genes chosen for RNAScope validation of Hb subpopulations. **A**) (**i**) *ONECUT2, TLE2*, *SEMA3D* marker gene expression *in situ*, with each detected cell object in the tissue section plotted as a gray or black rectangle. Cells in black have > 10 transcript copies of the Hb-wide marker gene *POU4F1*, indicating the presence of habenula. Colored circles mark the spatial location of the top 100 cells that most robustly express each marker gene. If a cell was ranked in the top 100 for more than one marker gene (see confusion matrices in Supplementary Figure 6B), it was colored by the gene for which it had the most number of transcript copies. (**ii**) RNAScope images of example top ranked cells that highly express *ONECUT2, TLE2*, *SEMA3D* at high magnification. **B)** (**i**) Spatial plots as described in **A** for *ESRP1*, *MCOLN3*, *CRH* marker gene expression *in situ*. (**ii**) RNAScope images of example top ranked cells that highly express *ESRP1*, *MCOLN3*, *CRH* at high magnification. **C)** (**i**) Spatial plots as described in **A** for *CCK*, *EBF3*, *CHAT* marker gene expression in situ. (**ii**) RNAScope images of example top ranked cells that highly express *CCK*, *EBF3*, *CHAT* at high magnification.

Given that rodent LHb and MHb neuronal subpopulations display topography ^23,24^, we validated the spatial organization of select human Hb subpopulations using single molecule fluorescent *in situ* hybridization (smFISH; n=2–3 donors per experiment; ETable 4, ETable 5). We used three probe combinations targeting 4 mean ratio marker genes each to visualize the following subpopulations (many of which could be identified by one marker gene, or a combination of 2 genes): LHb.1, LH.4, and LHb.5/1 (*ONECUT2*, *TLE2*, *SEMA3D*); LHb.2, LHb.3, and LHb.6 (*CRH*, *MCOLN3*, *ESRP1*); MHb.1, MHb.2, and MHb.3 (*CCK*, *CHAT*, *EBF3*) (Fig 3). Across donors, we observed spatial biases in some human Hb cell type locations (Supplementary Figure 6–8). For example, cells that highly expressed *ESRP1* (putative LHb.6 neurons) displayed a dorsal bias (Fig 3B). Furthermore, cells that highly expressed *CHAT* (putative MHb.2 neurons) clustered separately from other MHb cell types (Fig 3C). Taken together, smFISH results validated human Hb neuronal subpopulations identified by snRNA-seq, and corroborated findings in mice that molecularly defined LHb and MHb subpopulations show distinct spatial organization.

### UNIQUE SCHIZOPHRENIA-ASSOCIATED TRANSCRIPTOMIC CHANGES IN HUMAN HB COMPARED TO OTHER BRAIN REGIONS

**Figure 4:**
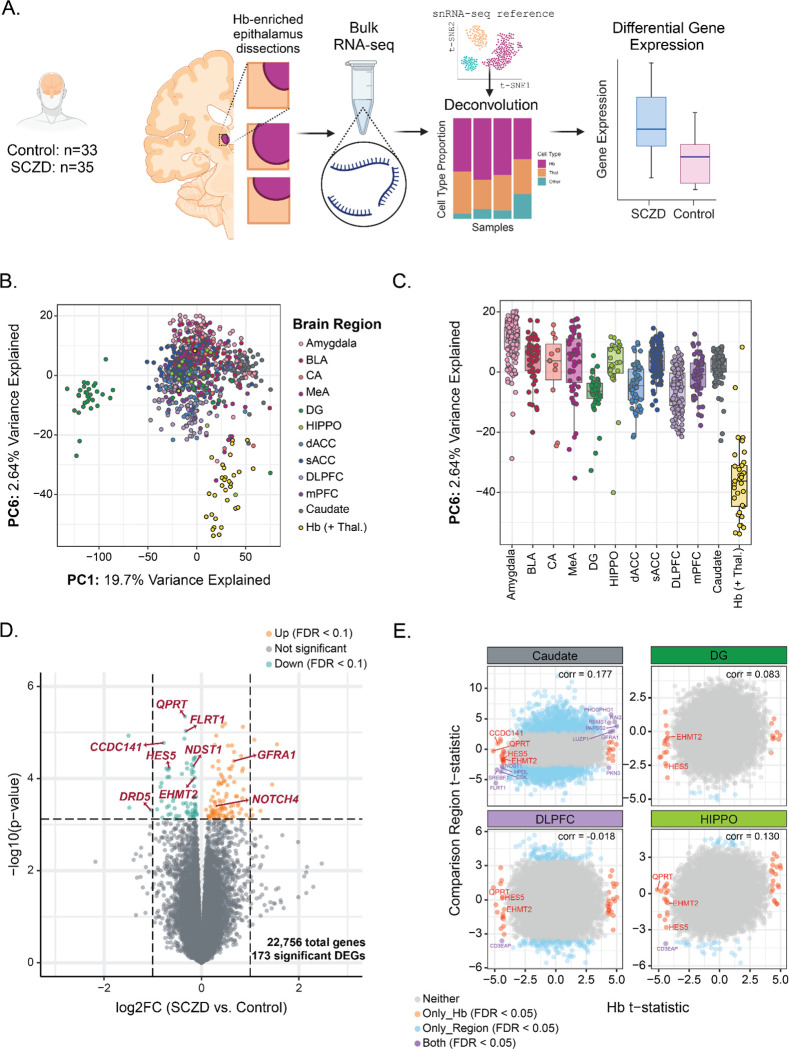
Identification of unique differentially expressed genes (DEGs) in Hb-enriched thalamus of Schizophrenia (SCZD) vs. Control cases. **A)** Study design and cell type deconvolution of postmortem human bulk RNA-seq samples using Hb single nucleus RNA-seq data (see Fig 1 and Supplementary Figure 12). **B)** Principal component analysis of postmortem human bulk RNA-seq data from neurotypical control tissue samples of Hb-enriched thalamus [Hb (+Thal.)] and 11 other brain regions (combined n = 817): amygdala, basolateral amygdala (BLA), cornu ammonis (CA), caudate, dorsal anterior cingulate cortex (dACC), dentate gyrus (DG), dorsolateral prefrontal cortex (DLPFC), hippocampus (HIPPO), medial amygdala (MeA), medial prefrontal cortex (mPFC), and subgenual anterior cingulate cortex (sACC). PC1 vs. PC6 scatter plot depicts PC6 separating Hb (+Thal.) from other regions. **C)** Boxplot of PC6 across 12 tested brain regions. **D)** Volcano plot of DGE analysis showing. upregulated genes (FDR < 0.1, orange points) and downregulated genes (FDR < 0.1, green points) in SCZD vs. Control cases. Horizontal dashed line demarcates the y-axis value corresponding to the significance threshold FDR = 0.1. Vertical dashed lines demarcate log2 fold change values of −1 and 1. **E)** SCZD vs. Control DEG comparison between Hb (+Thal.) and four other brain regions: Caudate, DG, HIPPO, and DLPFC. Only genes expressed in the Hb were considered in the comparative analysis. X and Y axes are *t*-statistic values. Red points are unique DEGs (FDR < 0.05) in Hb (+Thal.) compared to other brain regions tested. Blue points are DEGs (FDR < 0.05) found in comparison regions, but not Hb (+Thal.). Purple points are DEGs (FDR < 0.05) that overlap between Hb (+Thal.) and comparison regions.

To investigate transcriptomic changes associated with SCZD diagnosis in the human Hb, we performed bulk RNA-sequencing of postmortem human Hb-enriched tissue samples from 35 SCZD cases and 34 neurotypical controls (Fig 4A, ETable 1, Supplementary Figure 10). Due to inclusion of surrounding thalamic tissue during epithalamus dissections, we leveraged our human Hb snRNA-seq dataset (Fig 1) to implement cell type deconvolution of the bulk RNA-seq data. Samples contained some proportion of Hb cell types as estimated by utilizing the top 25 mean ratio marker genes for broad cell types (Supplementary Figure 12, Supplementary Figure 11, Supplementary Figure 16, ETable 6). The one control sample that did not meet this criterion was dropped from further analyses.

Prior to performing differential gene expression (DGE) analysis, we assessed how the transcriptomic landscape of our Hb-enriched samples compared to that of other brain regions and subregions implicated in psychiatric disorders. We conducted principal component analysis (PCA) on bulk RNA-seq data (subsetted to neurotypical control samples) from eleven regions (n = 817 total samples), including the amygdala, hippocampus, caudate, and several cortical areas (Fig 4B-C). Hb-enriched samples separated from other brain regions’ samples along the sixth principal component axis (PC6), which explained 2.64% of the variance in the combined dataset, highlighting that Hb-enriched thalamus has unique transcriptional features compared to other brain regions.

We then performed DGE analysis by diagnosis (SCZD vs. Control samples) to identify genes significantly upregulated or downregulated (FDR < 0.1) in Hb-enriched tissue (Fig 4D; ETable 7). We controlled for effects of covariates including age, estimated proportions of Hb and thalamic cells in the samples, bulk RNA-seq quality control metrics, and quality surrogate variables associated with RNA degradation ^55,56^, all of which explained a portion of the gene expression variance (Supplementary Methods, Supplementary Figure 17). We found 173 differentially expressed genes (DEGs, FDR < 0.1) between SCZD cases and controls, including several genes previously implicated in SCZD, such as *NOTCH4* (Fig 4D) ^37,68–71^. To evaluate SCZD-associated gene expression differences unique to Hb-enriched tissue compared to other brain regions, we conducted a cross-region comparison of DEGs obtained from our Hb-enriched dataset (n=45 DEGs, FDR < 0.05) and those from previously published bulk RNA-seq studies of four other brain regions: dorsolateral prefrontal cortex (DLPFC), hippocampus, dentate gyrus, and caudate ^19–21^ (Fig 4E). We found the highest number of overlapping DEGs (n=12, FDR < 0.05) with the caudate, with one or less overlapping DEGs in other brain regions tested. *PHOSPHO1*, an upregulated DEG in both Hb-enriched samples and caudate, showed enrichment in the human LHb.3 subpopulation (enrichment *t*-stat = 4.415). In summary, we identified 32 DEGs (FDR < 0.05) between Control and SCZD samples that were unique to the Hb-enriched dataset compared to other brain regions. These results support a critical role for the human Hb in neuropsychiatric disease, and provide potential Hb-specific molecular targets for functional follow up and therapeutic applications.

## Discussion

SCZD is a heritable polygenic disorder characterized by positive and negative symptoms, with heterogeneity in clinical presentation and treatment response ^72^. Therefore, it is important to understand how molecular changes in multiple brain regions contribute to SCZD etiology. Here, we focused on the Hb given its emerging role in psychiatric disorders and functional influence on neurotransmitter systems impacted in SCZD. To provide a basis for interpreting molecular changes associated with SCZD, we generated a topographic molecular map characterizing MHb and LHb cell type diversity in the healthy human brain. For instance, we identified a spatially-organized cholinergic cell type (MHb.2), which was distinguished from other *CHRNB4*-expressing MHb subpopulations by enrichment of *CHAT* and *CHRNA6*. This cholinergic subpopulation, which is conserved across species, may represent a shared biological substrate for comorbid nicotine dependence and SCZD ^73–77^. Beyond classifying cell types in the human Hb, we also leveraged our reference atlas to disentangle molecular signatures from Hb and thalamus in a bulk RNA-sequencing study comparing SCZD and control donors.

Differential gene expression analysis of bulk RNA-seq data from individuals with SCZD and controls identified 32 differentially expressed genes (DEGs) unique to Hb-enriched tissue compared to other brain regions. Among unique DEGs, we identified downregulation of *HES5* (FDR < 0.05), a target of Notch signaling highly expressed in neural stem cells (NSC) and implicated in the regulation of early-stage neurodevelopment. While Notch signaling is important for NSC fate, it is also implicated in neuroglial development ^68^. Interestingly, *HES5* is enriched in astrocytes (Fig 1; enrichment t-stat = 7.794), suggesting this glial population in epithalamus may be affected in SCZD. In a mouse model of SCZD, *HES5* deficiency in cultured hippocampal cells affected the proliferative capacity of uncommitted progenitors, suggesting that *HES5* may also have important developmental functions in neurons ^78^. Unique to Hb-enriched tissue, we also observed downregulation of dopamine receptor *DRD5* (FDR < 0.1). While little is known about the role of *DRD5* in SCZD, *DRD5* disruption in some animal models, but not others, is associated with cognitive deficits ^79–81^ and altered cortical oscillations ^82^. Given the role of dopamine signaling in SCZD pathogenesis and treatment, future functional follow up studies should explore how *DRD5* signaling is altered in the Hb in the context of SCZD.

Cross-region comparison of SCZD-associated DEGs in Hb-enriched tissue with those identified in the caudate, DLPFC, HIPPO, and dentate gyrus (DG) revealed the most overlap with caudate DEGs. For instance, *NOTCH4* was upregulated in both Hb-enriched and caudate SCZD samples. *NOTCH4* is associated with genetic susceptibility to SCZD ^37,69–71^, and plays a key role in neural maturation and plasticity. Consistent with the neurodevelopmental hypothesis of SCZD etiology ^39^, *Notch4* knockdown in a mouse NSC model leads to aberrations in cell proliferation, differentiation, and migration ^37–40^. We also identified overlapping upregulation of *PHOSPHO1* in the Hb and caudate datasets. *PHOSPHO1* encodes a phosphatase that hydrolyzes phosphocholine to choline*.* Given that choline is necessary for synthesizing the neurotransmitter acetylcholine, this finding may suggest that cholinergic neurons and cell populations expressing cholinergic receptors, particularly MHb.2, are more vulnerable to SCZD pathology. Furthermore, *PHOSPHO1* is regulated by a non-coding RNA (miR1306) in the chromosomal locus 22q11.2 ^83^. Microdeletions in this locus cause DiGeorge syndrome and lead to cognitive and behavioral impairments, as well as a strikingly elevated risk of developing SCZD or schizoaffective disorder ^84–87^. Further exploration of how PHOSPHO1 may be dysregulated in SCZD is warranted.

### Strengths and Limitations

Here we conduct the first transcriptomic study of postmortem human Hb in the context of SCZD. As with all postmortem human brain studies, we acknowledge our inability to confirm whether gene expression changes are the cause or consequence of disease. We tried to overcome some limitations by accounting for potential confounds related to donor demographics and RNA quality in our statistical model. We were also able to overcome challenges in precisely dissecting Hb tissue by performing cell type deconvolution of bulk RNA-seq data to control for thalamic contamination. Finally, we acknowledge that our study is limited to a small number of donors, including only Caucasian males, and that it will be important to replicate these findings in additional cohorts. In summary, we generated the first single cell molecular atlas of the human Hb and used this reference dataset to better understand gene expression changes associated with habenular dysfunction in SCZD.

## Conclusions

This study characterized cell type diversity in the human Hb and identified both molecularly-defined and spatially-organized lateral and medial neuronal populations. Transcriptomic analysis of Hb-enriched samples from individuals with SCZD and neurotypical controls revealed both shared and unique molecular changes in Hb compared to other brain regions, providing further support that the Hb is a promising therapeutic target for treating neuropsychiatric disorders.

## Supplementary Material

Supplement 1

Supplement 2

## Data Availability

The source FASTQ files are publicly available from the Globus endpoints ‘jhpce#habenulaPilotbulkRNAseq’, ‘jhpce#habenulaPilotsnRNAseq’, and ‘jhpce#habenulaPilotRNAscope’ for the bulk RNA-seq, snRNA-seq, and RNAScope data, respectively. The DNA genotype data is available from ‘jhpce#habenulaPilotbulkDNAgenotype’ available upon request, given the protected nature of this data. All these Globus endpoints are listed at http://research.libd.org/globus. All source code developed for analyzing this data is available at https://github.com/LieberInstitute/Habenula_Pilot ^88^.

## Key Points

### Question:

What is the molecular and cellular organization of the human habenula and how is it uniquely disrupted in schizophrenia?

### Findings:

In this single cell and spatial transcriptomic study of postmortem human habenula, we identified 10 molecularly defined medial and lateral habenula cell types. Several of these subpopulations were topographically organized and conserved across species. Bulk RNA-sequencing and cell type deconvolution of habenula-enriched tissue samples from 35 subjects with schizophrenia and 33 neurotypical controls revealed both shared and unique transcriptomic changes associated with schizophrenia compared to other brain regions.

### Meaning:

The results of this study identify molecular changes in the human epithalamus associated with schizophrenia, further justifying the habenula as a relevant therapeutic target for neuropsychiatric disorders.
