# Supplementary material for: Transcriptomic analysis of the human habenula in schizophrenia
Source: bioRxiv. 2024 Feb 27:2024.02.26.582081. Preprint. [Version 1] doi: 10.1101/2024.02.26.582081 (PMC10925152; doi:10.1101/2024.02.26.582081)
Supplement: Supplement 2 [file NIHPP2024.02.26.582081v1-supplement-2.pdf]

bioRxiv preprint doi: https://doi.org/10.1101/2024.02.26.582081; this version posted February 27, 2024. The copyright holder for this preprint (which was not certified by peer review) is the author/funder, who has granted bioRxiv a license to display the preprint in perpetuity. It is made available under aCC-BY 4.0 International license.

Supplemental Content

Supplemental Methods

Postmortem Human Clinical Characterization

Audiotaped and witnessed informed consent was obtained from the legal next-of-kin for every case. The LIBD Autopsy Phone Screening was performed at time of donation with the legal next-of-kin, and consisted of 39 items about the donor's medical, social, psychiatric, substance use, and treatment history. Retrospective clinical diagnostic reviews were conducted for every brain donor, which included data from autopsy reports, toxicology testing, forensic investigations, neuropathological examinations, phone screening, and psychiatric/substance abuse treatment record reviews and/or supplemental family informant interviews. All data was compiled in a detailed psychiatric narrative summary, and was reviewed independently by 2 board-certified psychiatrists to determine lifetime psychiatric and substance use disorder diagnoses according to DSM-5. Every donor underwent toxicology testing by the medical examiner as part of the autopsy and forensic investigation for drugs of abuse such as ethanol/volatiles, cocaine/metabolites, amphetamines, and opiates. Additional supplemental toxicology testing was performed on all donors, including for nicotine/cotinine and cannabinoids. For psychiatric cases, supplemental testing for therapeutic drugs, such as antidepressants, mood stabilizing agents, and antipsychotics was completed through National Medical Services (www.nmslabs.com) in postmortem blood and/or cerebellar tissue. All non-psychiatric control donors had no lifetime history of a psychiatric or substance use disorder according to DSM-5.

snRNA-seq Data Collection

Fresh frozen postmortem human brain tissue from seven adult neurotypical control male donors were dissected for epithalamus (Br1092, Br1204, Br1469, Br1735, Br5555, Br5558, and Br5639; see **ETable 1** for demographics and other information). Tissue was homogenized and nuclei isolation was performed using the "Frankenstein" protocol as previously described [42]. All samples were stained with propidium iodide (Cat No. P3566, Invitrogen, Thermo Fisher, Waltham, MA). Additionally, four of the seven samples (Br1092, Br1204, Br5555, Br5558) were stained with Alexa Fluor 488-conjugated Anti-NeuN (Cat No. MAB377X, Millipore Sigma, St. Louis, MO) to enrich for neuronal nuclei during fluorescence-activated nuclei sorting (FANS). Using a BioRad S3e cell sorter, nuclei were sorted into 23.1 uL of master mix without enzyme C, prepared as per the 3' next GEM Chromium Kit protocol (PN-1000075, 10x Genomics, Pleasanton, CA). Following FANS, GEMs were produced, cDNA was generated, and library preparations were completed by following revision A of the Chromium Next GEM Single Cell 3' Reagent Kits v3.1 (Dual Index) protocol (PN-1000075, PN-1000073, CG000315, 10x Genomics). Samples were sequenced on an Illumina platform according to manufacturer's instructions at the John Hopkins Single Cell and Transcriptomics Core.

bioRxiv preprint doi: https://doi.org/10.1101/2024.02.26.582081; this version posted February 27, 2024. The copyright holder for this preprint (which was not certified by peer review) is the author/funder, who has granted bioRxiv a license to display the preprint in perpetuity. It is made available under aCC-BY 4.0 International license.

snRNA-seq Quality Control, Clustering, and Annotation

snRNA-seq samples were sequenced to a median depth of 193 million reads (min. 162.8, mean 188.3, max. 213), corresponding to a median 54,641 mean reads per nucleus (min. 39,476, mean 88,719, max. 230,795), a median of 14,135 median unique molecular indices (UMIs) per nucleus (min. 7,752, mean 17,674, max. 34,479), and a median 4,592 median genes per nucleus (min. 2,836, mean 5,083, max. 7,843). FASTQ files were aligned with *CellRanger* v6.0.0 (https://www.10xgenomics.com/support/software/cell-ranger/latest) against the `refdata-gex-GRCh38-2020-A` annotation distributed by 10x Genomics. The `--include-introns` option was used. Prior to quality control processing, there were 20,327 nuclei across all seven samples with a median of 3,156 nuclei per sample (min. 923, mean 2,904, max. 4,389) as estimated by *CellRanger* (**ETable 2**).

Starting from the raw data instead of the *CellRanger* filtered data, empty droplets were excluded using *DropletUtils* v1.18.1 [43] with `emptyDrops(niters = 30000, lower = knee_lower)`, where `knee_lower` was determined for each sample by the "knee point" calculated by *DropletUtils* `barcodeRanks()` function plus 100. This resulted in `knee_lower` values ranging from 219 to 481. Droplets with a significant deviation from each sample's ambient profile (FDR < 0.001) were kept, resulting in a dataset of 19,802 nuclei at this processing stage (936 to 3,905 per sample).

As part of quality control, nuclei were assessed for high mitochondrial content, low library size, and a low number of detected features. We applied an adapted 3 median absolute deviation (MAD) threshold per sample using *scater* v1.26.1 `isOutlier(nmads=3)` [45] (**Supplementary Figure 1**). Any nuclei that did not pass each threshold were excluded from further analyses (2,720 nuclei), resulting in 17,082 nuclei that passed this stage of quality control. Lastly, doublet scores were computed per sample using the top 1,000 highly variable genes with *scDblFinder* v1.12.0 `computerDoubletDensity()` [89]. This metric was later leveraged to assess the integrity of each cell type cluster. No cell type clusters were dropped as a result of their doublet scores.

Dimension reduction was performed using the generalized linear model for principal component analysis (GLM-PCA), using both *scry* v1.10.0 `nullResiduals()` [90] and *scater* v1.26.1 `runPCA()` [45]. It was apparent that the initial reduced dimensions showed evidence for batching by `Sample`, sequencing run, and NeuN sorting **(Supplementary Figure 3A, Supplementary Figure 4A, Supplementary Figure 5A)**. To correct for these batch effects, we applied *Harmony* v0.1.1 `RunHarmony(group.by.vars = "Sample")` [44] to the GLM approximated PCs. This returned Harmony-corrected PCs, which showed evidence of successfully correcting for differences across the aforementioned variables **(Supplementary Figure 3B, Supplementary Figure 4B, Supplementary Figure 5B)**.

After correcting by `Sample`, we applied graph-based clustering measures leveraging *scran* v1.26.2 [46] `buildSNNGraph(k = 10)` and *igraph* v1.4.2 `cluster_walktrap()` [91],

bioRxiv preprint doi: https://doi.org/10.1101/2024.02.26.582081; this version posted February 27, 2024. The copyright holder for this preprint (which was not certified by peer review) is the author/funder, who has granted bioRxiv a license to display the preprint in perpetuity. It is made available under aCC-BY 4.0 International license.

generating 37 fine-resolution nuclei clusters. Using established marker genes [23], we annotated our 37 fine-resolution nuclei clusters for cell type identities. This process yielded 17 cell type categories that maintained the distinct Hb subclusters while collapsing all other non-Hb clusters into their respective cell type populations (**Fig 1B**): 3 Astrocyte clusters, 11 Excitatory Thalamus clusters, 5 Inhibitory Thalamus clusters, and 3 Oligodendrocyte clusters were collapsed. We were thus ultimately left with 7 broad cell type clusters (i.e. Oligodendrocytes, OPC, Microglia, Astrocytes, Endothelial, Inhibitory Thalamus, and Excitatory Thalamus) alongside our 7 Lateral Habenula (LHb) and 3 Medial Habenula (MHb) cell type subclusters.

No given cell type cluster was driven by doublet scoring as no cluster had a particularly high median doublet score (**Supplementary Figure 2A**). Out of the 17,082 nuclei post-`isOutlier()` filters, one small cluster (51 nuclei) did not have a clear cell type identity, but did have high expression of *SNAP25*. This small cluster was thus classified as "Excit.Neuron" and excluded from downstream analyses (**Supplementary Figure 2**). Nuclei from three donors (Br5555, Br1204, Br1092) in the OPC cluster were labeled as "OPC_noisy" (594 nuclei) given their less defined spatial arrangement in *t*-SNE dimensions 1 and 2, and were excluded from further analyses. The entire quality control, clustering, and annotation processes brought the initial number of 20,327 nuclei to a final total of 16,437 filtered and annotated nuclei (**Supplementary Figure 13**).

snRNA-seq Marker Gene Selection

Marker genes were selected for the 17 identified cell type categories through the *Mean Ratio* method from *DeconvoBuddies* v0.99.0 (https://github.com/LieberInstitute/DeconvoBuddies), using the function `getMeanRatio2()`. The mean ratio method calculates, for each gene, the mean expression of a target cell type divided by the highest mean expression of a non-target cell type. A high mean ratio value for a gene in a given cell type cluster suggests that that gene is a cell type specific marker gene candidate for that cell type cluster. The top 50 mean ratio marker genes for each of our cell type categories are listed in a table (**ETable 3**).

Cross-species Comparison

Cell type clusters observed in the human snRNA-seq dataset were compared to a previously annotated single cell RNA-sequencing dataset from mouse habenula [22]. The mouse dataset had annotations across all cell types, and lateral and medial specific annotations for just the Hb neurons. To relate the mouse and human datasets, homologous gene IDs were found using the Mouse Gene Informatics Website. Both of the datasets were subset to 14,468 genes with valid homologs [92]. The two datasets were compared via spatial registration pipeline from *spatialLIBD* v1.12 [93], and gene enrichment statistics for each cell type in both annotations were calculated with `registration_wrapper()`. For a more targeted comparative analysis, we utilized a "neuron-only" mouse dataset, and in this case, enrichment statistics were computed on the "habenula neuron-only" subset of our human snRNA-seq dataset. In both iterations of the comparative analyses, correlation values between the *t*-statistics were calculated with

bioRxiv preprint doi: https://doi.org/10.1101/2024.02.26.582081; this version posted February 27, 2024. The copyright holder for this preprint (which was not certified by peer review) is the author/funder, who has granted bioRxiv a license to display the preprint in perpetuity. It is made available under aCC-BY 4.0 International license.

`layer_stat_cor(top_n = 100)` and heatmaps were plotted with *ComplexHeatmap* v2.16 [94] (**Fig 2A-B**).

Multiplexed Single molecule fluorescent *in situ* Hybridization (smFISH)

smFISH experiments were performed according to manufacturer's instructions as previously described using the RNAScope Multiplex Fluorescent Reagent Kit v2 (Advanced Cell Diagnostics, Hayward, California, Cat No. 323100) and 4-Plex Ancillary Kit for Multiplex Fluorescent Kit v2 (Cat No. 323120) [47]. Fresh frozen tissue blocks containing Hb from three independent donors (**ETable 4**) were cryosectioned at ~10 µm on a Leica cryostat and stored at -80°C. Each donor yielded 2-3 tissue slides with 2-4 tissue sections per slide. Slides were assigned to three different RNAScope experiments targeting different LHb and MHb cell type clusters based on results from snRNA-seq (**Fig 1**, **Supplementary Figure 6A**, **Supplementary Figure 7A**, **Supplementary Figure 8A**). Experiment 1 targeting LHb subclusters: *ONECUT2* (Cat No. 473531-C1), *TLE2* (Custom Design 1271611-C2), *SEMA3D* (Cat No. 521771-C3), and *POU4F1* (Cat No. 438441-C4). Experiment 2 LHb subclusters: *ESRP1* (Cat No. 435051-C1), *MCOLN3* (Cat No. 516761-C2), *CRH* (Cat No. 473661-C3), and *POU4F41* (Cat No. 438441-C4). Experiment 3 MHb subclusters: *CCK* (Cat No. 539041-C1), *POU4F1* (Cat No. 438441-C2), *EBF3* (Cat No. 581641-C3), and *CHAT* (Cat No. 450671-C4); or *CCK* (Cat No. 539041-C1), *CHRNB4* (Cat No. 482411-C2), *BHLHE22* (Cat No. 448351-C3), and *CHAT* (Cat No. 450671-C4)

Briefly, tissue sections were fixed in 10% Normal Buffered Formalin (NBF) solution for 30 minutes at room temperature. Sections were then rinsed in 1x phosphate buffered saline (PBS) and sequentially dehydrated for 5 minutes each in four ethanol dilutions: 50%, 75%, 100%, and 100%. Once the tissue sections were dry, a hydrophobic pen was used to carefully outline them on the slides to form a hydrophobic barrier. Tissue sections were then treated with hydrogen peroxide for 10 minutes at room temperature. Following subsequent decanting and rinsing in 1xPBS, tissue sections were permeabilized with Protease IV for 30 minutes at room temperature. After decanting and rinsing the tissue slides in 1xPBS, probe hybridization solution was prepared. Three different sets of RNAScope probe combinations (4 probes per combination) were used for each experiment as described above.

Tissue sections were incubated in probe hybridization solution at 40°C for 2 hours. Following decanting and wash steps in 1x wash buffer, sections were incubated in saline-sodium citrate buffer (SSC) overnight at 4°C. Next, probe signal amplification steps were performed and a distinct Opal fluorophore (520, 570, 620, 690 nm) was assigned to each probe (Perkin Elmer, Waltham, MA; 1:500) (**ETable 5**). Sections were counterstained with DAPI (4′,6-diamidino-2-phenylindole) to label nuclei. For each donor and probe combination, a 20X max-intensity projected z-stack image of the tissue section containing the largest habenula region (as gauged by signal in the *POU4F1* probe channel) was obtained with a Nikon AXR confocal microscope using spectral imaging and linear unmixing as previously described [47]. Confocal imaging data were saved as .nd2 files for downstream quantitative analysis.

bioRxiv preprint doi: https://doi.org/10.1101/2024.02.26.582081; this version posted February 27, 2024. The copyright holder for this preprint (which was not certified by peer review) is the author/funder, who has granted bioRxiv a license to display the preprint in perpetuity. It is made available under aCC-BY 4.0 International license.

smFISH Confocal Image Analysis using HALO

HALO (Indica Labs) was used to segment and quantify fluorescent signals for each probe in single cells. Nikon .nd2 files were imported into HALO and an analysis magnification value of 2 (corresponding to 40X for a 20X image) was chosen as this is the recommended magnification for punctate probe signal quantification. For each image, nuclear detection and smFISH probe detection parameters were optimized to minimize false positives by referencing the *HALO 3.6 User Guide* (Indica labs, February 2023). For example, contrast threshold, minimum signal intensity, size (min and max), roundness (min), and segmentation aggressiveness were determined for each probe as well as DAPI. All HALO settings files are available on Github at https://github.com/LieberInstitute/Habenula_Pilot/tree/master/processed-data/14_RNAscope/HALO_data [88].

The *FISH-IF* module was used to quantify RNA transcripts (copy counts) within each detected object (i.e. a nucleus with dilated boundary to estimate a “cell”) with reference to the manufacturer’s guidelines: *HALO 3.3 FISH-IF Step-by-Step guide* (Indica labs, v2.1.4 July 2021). Distributions of object signal intensity values across the 4 RNAScope probe channels were assessed to determine representative values for each channel’s copy intensity parameter. The copy intensity parameter determines how many RNA transcript copies are assigned to each cell. For each of the RNAScope probe channels, the median of the probe signal intensity values of cells that had non-zero signal in that particular channel was chosen as a representative/typical intensity value for that channel’s copy intensity parameter. Thorough comparisons between the raw image files and HALO analysis segmentation outputs were conducted to ensure accurate representation of the imaging data.

Visualization of Quantified smFISH Data

The spatial expression patterns of chosen probes as quantified by HALO were observed using hexbin plots displaying the maximum number of transcript copies in a bin generated with `stat_summary_hex(x = XMax, y = YMax, z = copies), fun = max, bins = 100)` from *ggplot2* v3.4 [95] (**Supplementary Figure 6C**, **Supplementary Figure 7C**, **Supplementary Figure 8C**). To focus on cell objects with robust expression of the marker gene probes, we visualized the top 100 cells ranked by number of transcript copies for each marker gene as a proxy for cell type. Note that ranks could include ties, in some cases leading to more than 100 cells being selected. As shown via confusion matrices, there was ~10% overlap between most of the top 100 assignments depending on the donor slide and marker gene pair being compared (**Supplementary Figure 6B**, **Supplementary Figure 7B**, **Supplementary Figure 8B**). To observe the relative spatial location of the top 100 cells for each marker gene, points colored by the marker gene (and related Hb subcluster) identity were plotted in the X-Y locations of the cell objects, over a background of black points identifying high expression of the established Hb-wide marker gene *POU4F1*. If a cell had more than one top 100 identity, its point color reflected the marker gene with the maximum number of transcript

bioRxiv preprint doi: https://doi.org/10.1101/2024.02.26.582081; this version posted February 27, 2024. The copyright holder for this preprint (which was not certified by peer review) is the author/funder, who has granted bioRxiv a license to display the preprint in perpetuity. It is made available under aCC-BY 4.0 International license.

copies in that cell (**Fig 3A-Ci**, **Supplementary Figure 6B**, **Supplementary Figure 7B**, **Supplementary Figure 8B**).

Bulk RNA-seq Data Collection

Total RNA was extracted from samples using the Qiagen AllPrep DNA/RNA/miRNA Universal Kit (Cat No./ID: 80224). Paired-end strand-specific sequencing libraries were prepared from 300 ng total RNA using the TruSeq Stranded Total RNA Library Preparation kit with Ribo-Zero Gold ribosomal RNA depletion (https://www.illumina.com/products/selection-tools/rrna-depletion-selection-guide.html) which removes rRNA and mtRNA. For quality control, synthetic External RNA Controls Consortium (ERCC) RNA Mix 1 (Thermo Fisher Scientific) was spiked into each sample. The libraries were sequenced on an Illumina HiSeq 3000 at the LIBD Sequencing Facility, producing from 30.14 to 645.8 million (median 90.65, mean 150.07) 100-bp paired-end reads per sample.

Bulk RNA-seq Data Processing

Bulk RNA-seq FASTQs were aligned to Gencode v25 [48] using *SPEAQeasy*'s [49] development version that consisted of SGE scripts (https://github.com/LieberInstitute/RNAseq-pipeline). The settings were: `--experiment "Roche_Habenula" --prefix "PairedEnd" --reference "hg38" --stranded "reverse" --ercc "TRUE"`. This resulted in 24.3 to 612.18 million reads mapped (median 80.53, mean 132.48) with an overall mapping rate (overallMapRate) of 0.5471 to 0.9169 per sample (median 0.8506, mean 0.8368). All *SPEAQeasy* metrics are available (**ETable 1**).

Deconvolution

Cell type deconvolution of the bulk RNA-seq data was performed with Bisque [52] from the R package *BisqueRNA* version 1.0.5, with the function `ReferenceBasedDecomposition(use.overlap = FALSE)`. The snRNA-seq data from the present study (**Fig 1**) was used as the reference dataset at the broad resolution; that is, by re-labeling MHb.1, MHb.2, MHb.3 as MHb, and similarly re-labeling the LHb fine-resolution clusters broadly as LHb. Across the broader-resolution clusters, marker genes were selected through the *Mean Ratio* method from *DeconvoBuddies* v0.99.0 (https://github.com/LieberInstitute/DeconvoBuddies) using the function `getMeanRatio2()`. The top 25 mean ratio marker genes for each cell type were selected as the marker genes for deconvolution (**ETable 6**). For comparative purposes, we also calculated the standard log2 Fold Change (std.logFC, **Supplementary Figure 11**) using `findMarkers(test="t", direction="up", pval.type="all")` from *scran* v1.26.2 [46] with a model adjusting for the donor ID and contrasting a given cell type against all the rest. The top 25 mean ratio marker genes typically have a high standard log2 fold change (**Supplementary Figure 11**, **ETable 6**) and can be visually inspected for validation (**Supplementary Figure 16**) using *iSEE* [57] interactive websites at https://github.com/LieberInstitute/Habenula_Pilot#interactive-websites.

bioRxiv preprint doi: https://doi.org/10.1101/2024.02.26.582081; this version posted February 27, 2024. The copyright holder for this preprint (which was not certified by peer review) is the author/funder, who has granted bioRxiv a license to display the preprint in perpetuity. It is made available under aCC-BY 4.0 International license.

Bulk RNA-seq Quality Control

Sample Br5772 resulted in an estimated 100% inhibitory thalamus proportion, and was dropped from further analyses (**Supplementary Figure 12**). *SPEAQeasy* [49] quality control metrics such as the mitochondrial mapping rate (mitoRate) and ribosomal RNA mapping rate (rRNA rate) were visually inspected across each flow cell (**Supplementary Figure 10**), as well as by schizophrenia (SCZD) case-control status. Outlier samples in principal component analyses did not seem to be related to any specific metrics, except for Br5572, which as noted previously had unusual deconvolution results. Contrasting the range of these *SPEAQeasy* metrics against other similar studies [19–21,53], the remaining 68 bulk RNA-seq samples were considered of appropriate quality for downstream analyses.

Bulk RNA-seq quality Surrogate Variables Calculation

To adjust for RNA degradation effects [55], we calculated quality surrogate variables (qSVs) with *qsvaR* [56]. We used `qSVA(type = "standard")` from *qsvaR* v1.5.3 on the filtered and normalized transcript data (TPM) with the model `~ PrimaryDx + AgeDeath + Flowcell + mitoRate + rRNA_rate + totalAssignedGene + RIN + abs_ERCCsumLogErr + tot.Hb + tot.Thal`, where `tot.Hb = LHb + MHb` and `tot.Thal = Inhib.Thal + Excit.Thal`. This resulted in 8 qSVs from 1,772 transcripts associated with RNA degradation in six brain regions as described in the *qsvaR* documentation (**ETable 1**). Note that habenula is not one of these six regions.

Bulk RNA-seq Variance Partition Across Genes

We used both *scater* and *variancePartition* to explore the percent of variance explained across all genes (**Supplementary Figure 17**) by different *SPEAQeasy* [49] metrics, sample demographic variables such as age at time of death, and qSVs (**ETable 1**). We ran `plotExplanatoryVariables()` from *scater* v1.28.0 [45] with default parameters. We also used `getVarianceExplained()`, `canCorPairs()`, and `fitExtractVarPartModel()` from *variancePartition* v1.30.2 [96] with the log normalized counts (`logcounts`). According to the *variancePartition* user guide, categorical variables such as `PrimaryDx` and `Flowcell` were treated as random effects.

Bulk RNA-seq Differential Expression Analysis

Gene level differential expression analysis was performed using `calcNormFactors()` from *edgeR* v3.42.4 [97] and `voom()`, `lmFit()`, `eBayes()`, and `topTable()` from *limma* v3.56.2 [54] (**ETable 7**). The design model was `~ PrimaryDx + AgeDeath + Flowcell + mitoRate + rRNA_rate + totalAssignedGene + RIN + abs_ERCCsumLogErr + tot.Hb + tot.Thal + qSV[1-8]`. This model tested for differences by SCZD case-control status stored in the primary diagnosis (`PrimaryDx`) variable. Differential expression results were visualized using *EnhancedVolcano* v1.18.0 [98].

bioRxiv preprint doi: https://doi.org/10.1101/2024.02.26.582081; this version posted February 27, 2024. The copyright holder for this preprint (which was not certified by peer review) is the author/funder, who has granted bioRxiv a license to display the preprint in perpetuity. It is made available under aCC-BY 4.0 International license.

Cross-Brain Region bulk RNA-seq Data Integration

Control bulk RNA-seq Hb-enriched samples were compared to bulk RNA-seq samples generated at the Lieber Institute for Brain Development from other brain regions with the same demographics: neurotypical individuals, adults at age of death (17-70 years), male, and european descent (EUR/CAUC).

- Amygdala: n = 140, (Moods) [53]
- Basolateral amygdala (BLA): n=54, VA_PTSD [99]
- Cornu Ammonis (CA): n=11, PTSD BrainOmics
- Caudate: n = 82, [20]
- Dorsal anterior cingulate cortex (dACC): n = 55, VA_PTSD [99]
- Dentate gyrus (DG): n = 41, DG Astellas [19]
- Dorsolateral prefrontal cortex (DLPFC): n = 121 (BSP2 + VA_PTSD) [21,99]
- Hippocampus (HIPPO): n = 26 (BSP2 + VA_PTSD) [21,99]
- Medial amygdala (MeA): n = 55, VA_PTSD [99]
- Medial prefrontal cortex (mPFC): n = 57, (BSP4+5 + PTSD BrainOmics)
- Subgenual anterior cingulate cortex (sACC): n = 142 (Moods) [53]

Principal components were computed using `prcomp()` on log2(RPKM + 1) expression values for genes with a mean RPKM > 0.1 across all brain regions. The percent of variance explained was computed with `getPcaVars()` from *jaffelab* v0.99.31 (https://github.com/LieberInstitute/jaffelab).

SCZD vs. Control Differential Gene Expression (DGE) Signal Comparison Across Brain Regions

We downloaded the schizophrenia vs. control DGE results from the BrainSEQ Phase II dorsolateral prefrontal cortex (DLPFC) and hippocampus (HIPPO) [21], BrainSEQ Phase III caudate [20], and dentate gyrus (DG) bulk RNA-seq datasets [19]. All of the above analysis results were generated at the Lieber Institute for Brain Development. We compared these results with the SCZD vs. Control DGE results from our Hb-enriched dataset by subsetting the BrainSEQ and DG datasets to only include genes that were expressed in Hb (**ETable 7**, **Fig 4E**).

Only 1 out of 236 DLPFC, 1 out of 46 HIPPO, 0 out of 10 DG, and 12 out of 2,437 caudate DEGs (FDR < 5%) were also significantly differentially expressed in Hb (FDR < 5%).

Software

ggplot2 v3.4.2 and earlier versions [95], *R* versions 4.1, 4.2, and 4.3 [100] , and *Bioconductor* versions 3.14, 3.16, and 3.18 [101] were used for the analyses.

bioRxiv preprint doi: https://doi.org/10.1101/2024.02.26.582081; this version posted February 27, 2024. The copyright holder for this preprint (which was not certified by peer review) is the author/funder, who has granted bioRxiv a license to display the preprint in perpetuity. It is made available under aCC-BY 4.0 International license.

Supplementary Figures

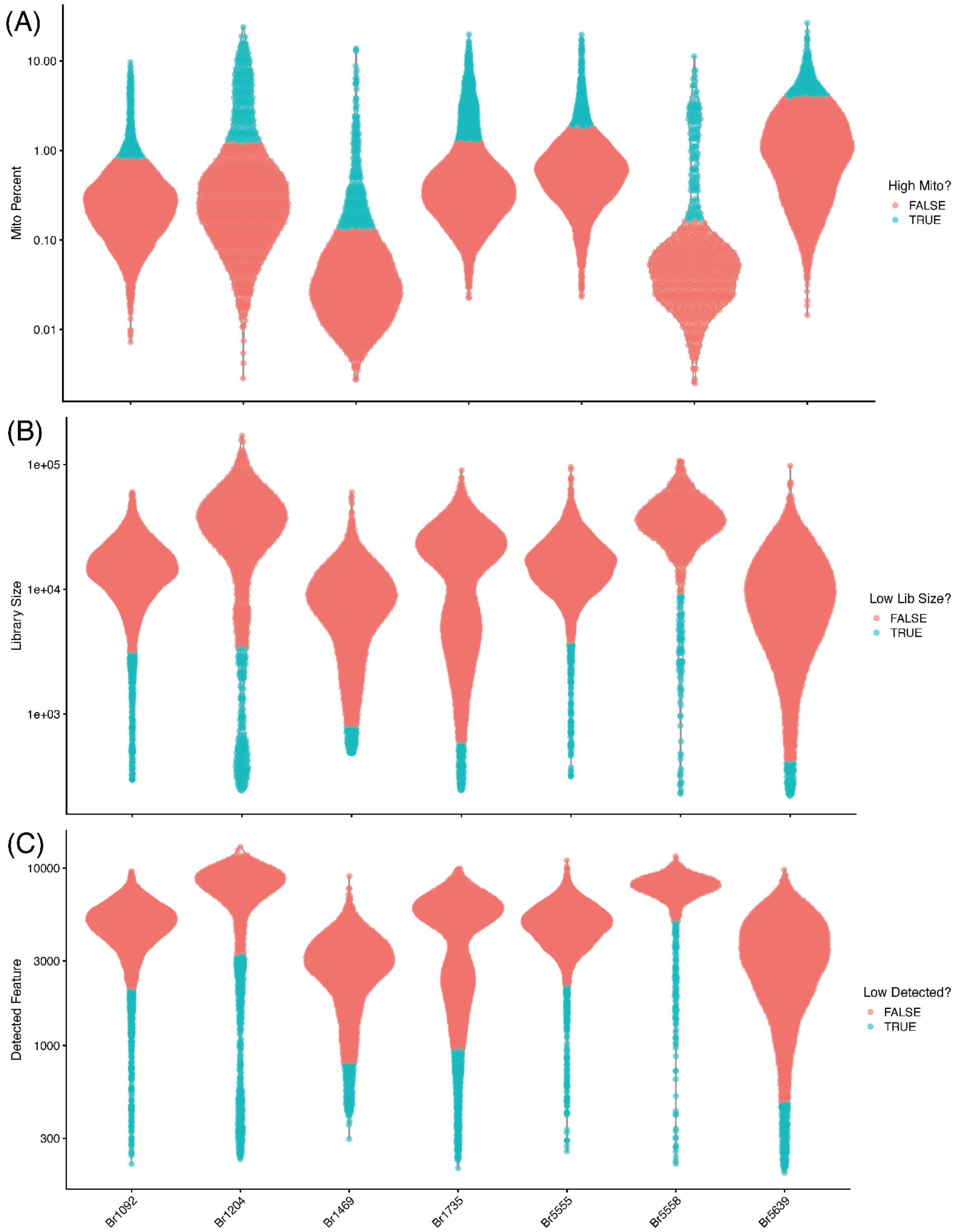

Supplementary Figure 1: snRNA-seq quality control metrics. Violin distribution plots for **A**) mitochondrial percent, **B**) library size (total number of UMIs), and **C**) number of detected genes. Thresholds for quality control were determined using `isOutlier()` from *scuttle*.

bioRxiv preprint doi: https://doi.org/10.1101/2024.02.26.582081; this version posted February 27, 2024. The copyright holder for this preprint (which was not certified by peer review) is the author/funder, who has granted bioRxiv a license to display the preprint in perpetuity. It is made available under aCC-BY 4.0 International license.

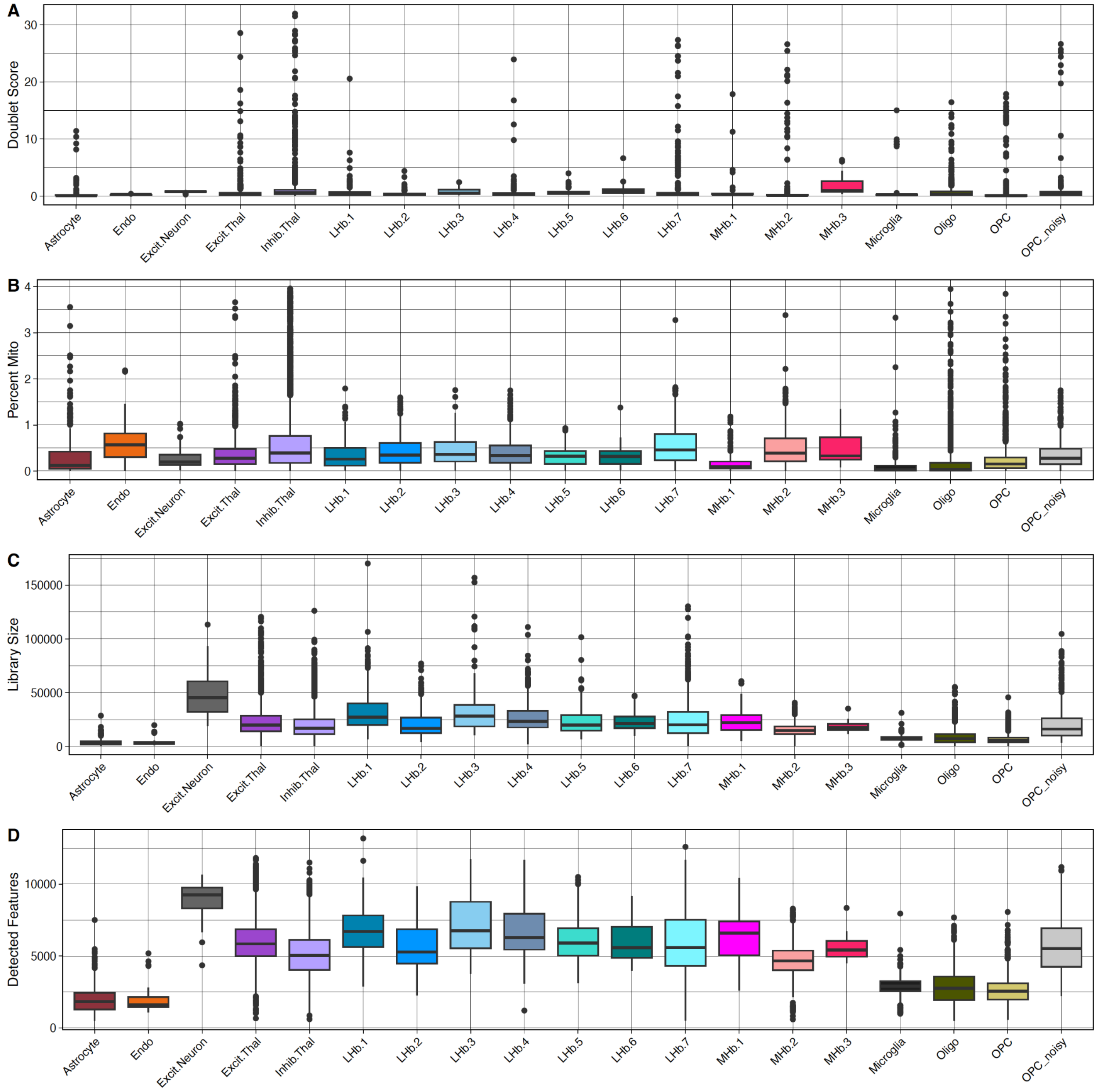

Supplementary Figure 2: snRNA-seq quality control metrics by cell type. Boxplot distribution plots for each identified cell type looking at **A**) doublet score, **B**) mitochondrial percent, **C**) library size (total number of UMIs), and **D**) number of detected genes. Ambiguous excitatory neurons (Excit.Neuron) and OPCs (OPC_noisy) were dropped from further analyses.

bioRxiv preprint doi: https://doi.org/10.1101/2024.02.26.582081; this version posted February 27, 2024. The copyright holder for this preprint (which was not certified by peer review) is the author/funder, who has granted bioRxiv a license to display the preprint in perpetuity. It is made available under aCC-BY 4.0 International license.

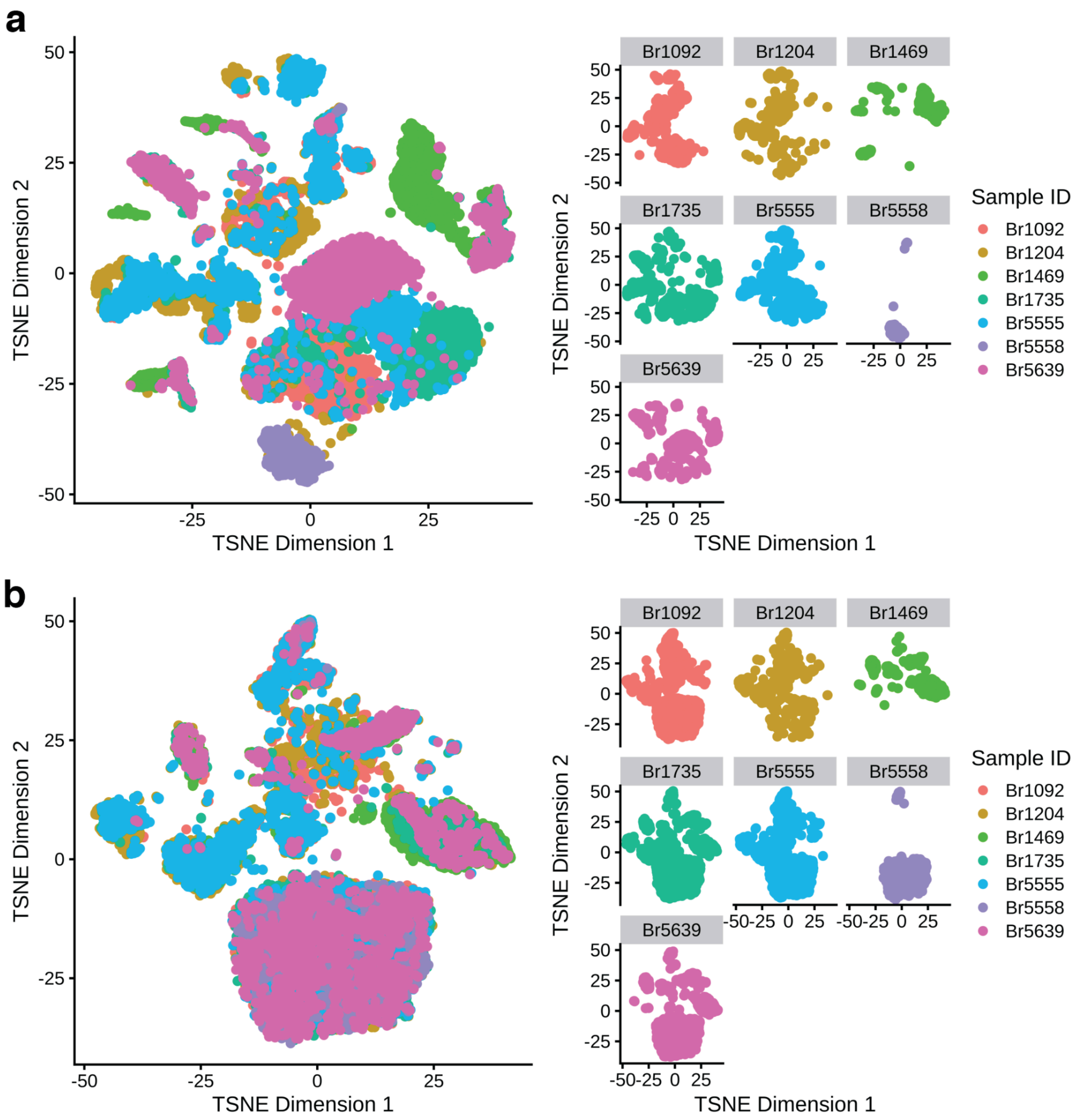

Supplementary Figure 3: Pre and post harmony by sample. Batch correction of snRNA-seq data visualized by t-SNE plots. **A**) t-SNE of principal components (PCs) pre-batch correction, colored by sample. **B**) t-SNE of PCs post-batch correction with *Harmony*, colored by sample. Batch correction with *Harmony* reduced the sample batch effect.

bioRxiv preprint doi: https://doi.org/10.1101/2024.02.26.582081; this version posted February 27, 2024. The copyright holder for this preprint (which was not certified by peer review) is the author/funder, who has granted bioRxiv a license to display the preprint in perpetuity. It is made available under aCC-BY 4.0 International license.

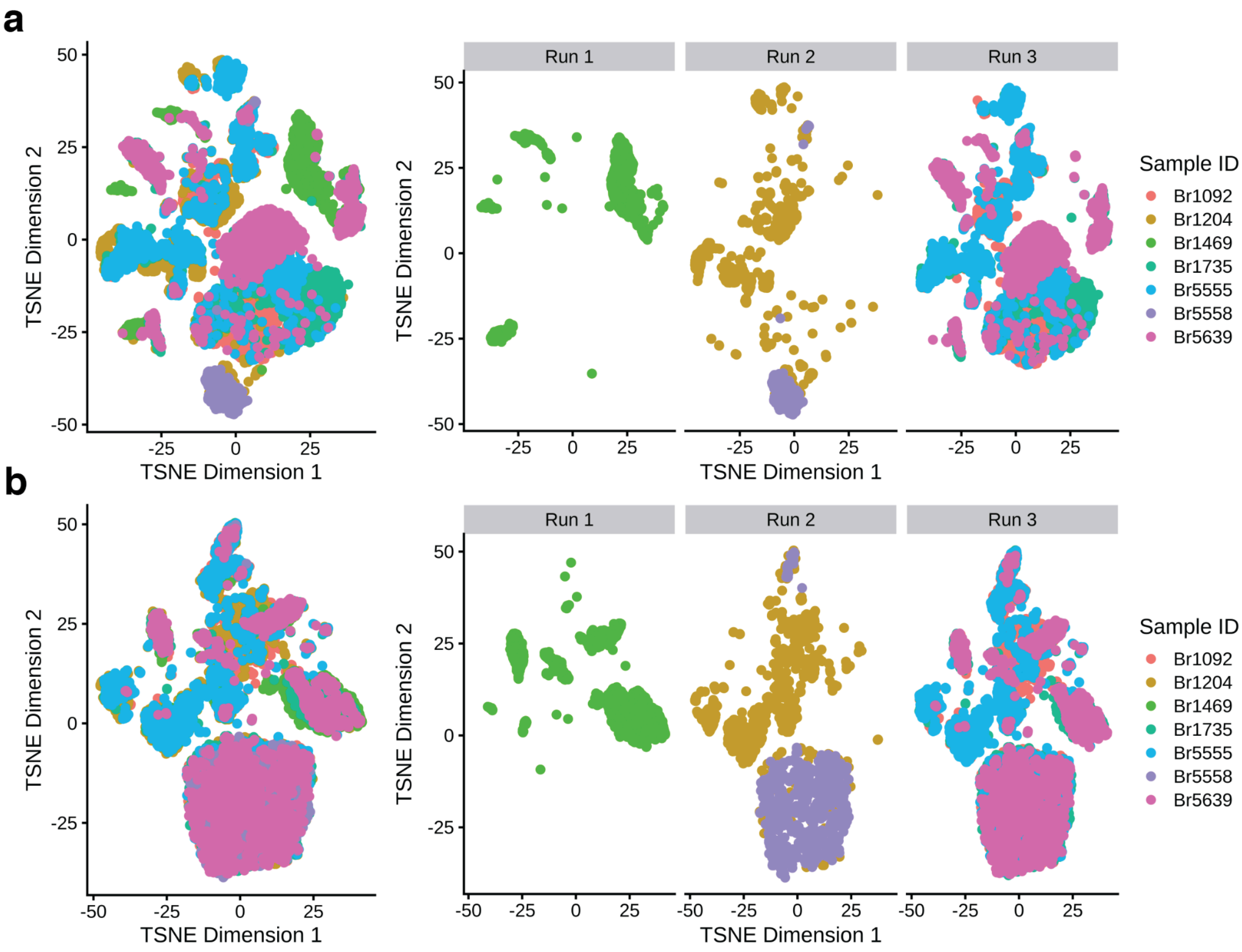

Supplementary Figure 4: Pre and post harmony by sequencing run. Batch correction of snRNA-seq data visualized by t-SNE plots. **A**) t-SNE of principal components (PCs) pre-batch correction, colored by sample and faceted by sequencing run. **B**) t-SNE of PCs post-batch correction with *Harmony*, colored by sample and faceted by sequencing run. Batch correction with *Harmony* reduced sample and sequencing run batch effects.

bioRxiv preprint doi: https://doi.org/10.1101/2024.02.26.582081; this version posted February 27, 2024. The copyright holder for this preprint (which was not certified by peer review) is the author/funder, who has granted bioRxiv a license to display the preprint in perpetuity. It is made available under aCC-BY 4.0 International license.

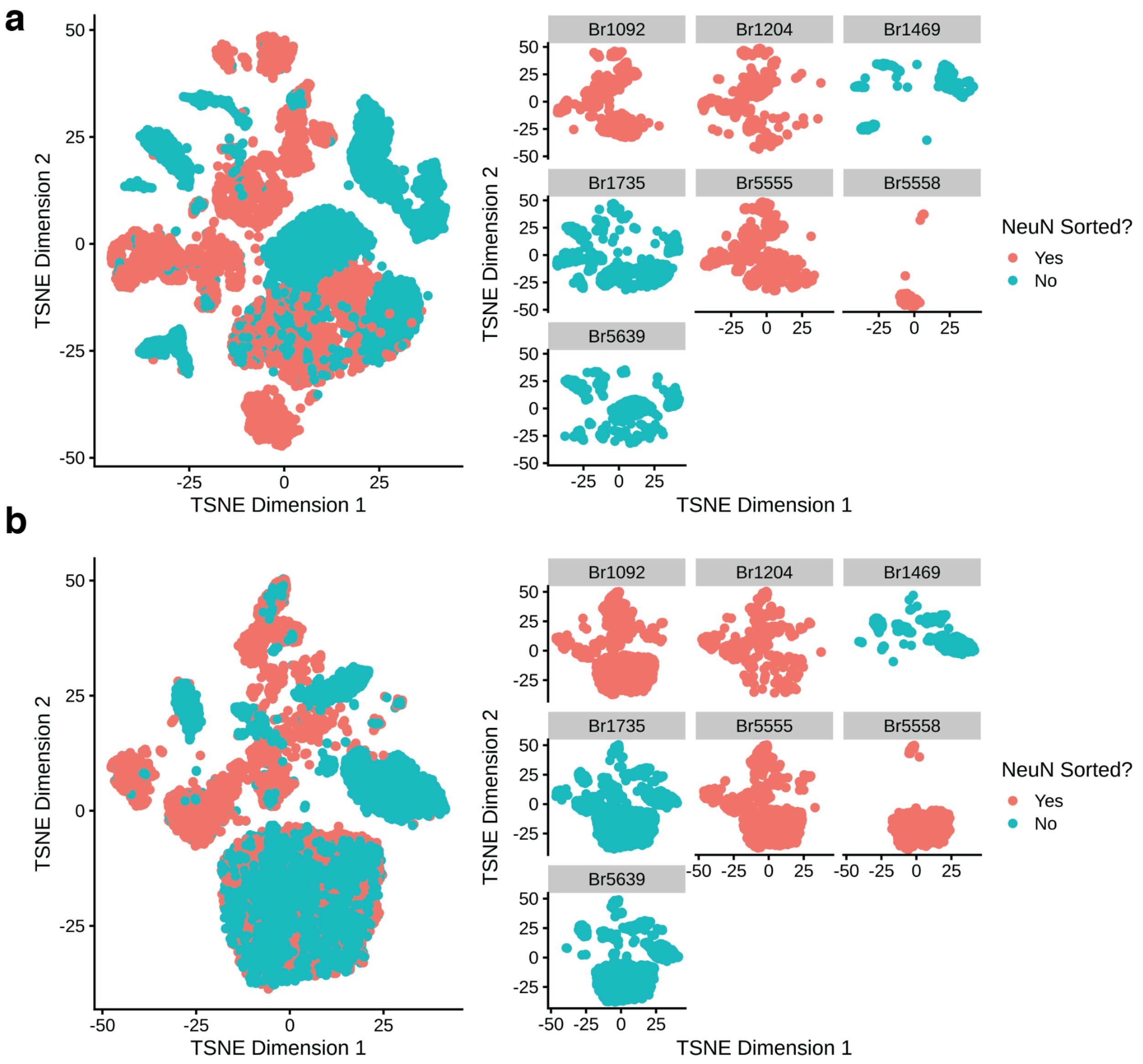

Supplementary Figure 5: Pre and post harmony by NeuN sorting. Batch correction of snRNA-seq data visualized by t-SNE plots. **A**) t-SNE of principal components (PCs) pre-batch correction, colored by neuronal enrichment performed with NeuN antibody labeling and FANS. **B**) t-SNE of PCs post-batch correction with *Harmony*, colored by neuronal enrichment performed with NeuN antibody labeling and FANS. While batch correction with *Harmony* reduced NeuN sorting differences, some differences remain. This is expected given that the NeuN sorted samples are enriched for neuronal cell types compared to samples without NeuN sorting.

bioRxiv preprint doi: https://doi.org/10.1101/2024.02.26.582081; this version posted February 27, 2024. The copyright holder for this preprint (which was not certified by peer review) is the author/funder, who has granted bioRxiv a license to display the preprint in perpetuity. It is made available under aCC-BY 4.0 International license.

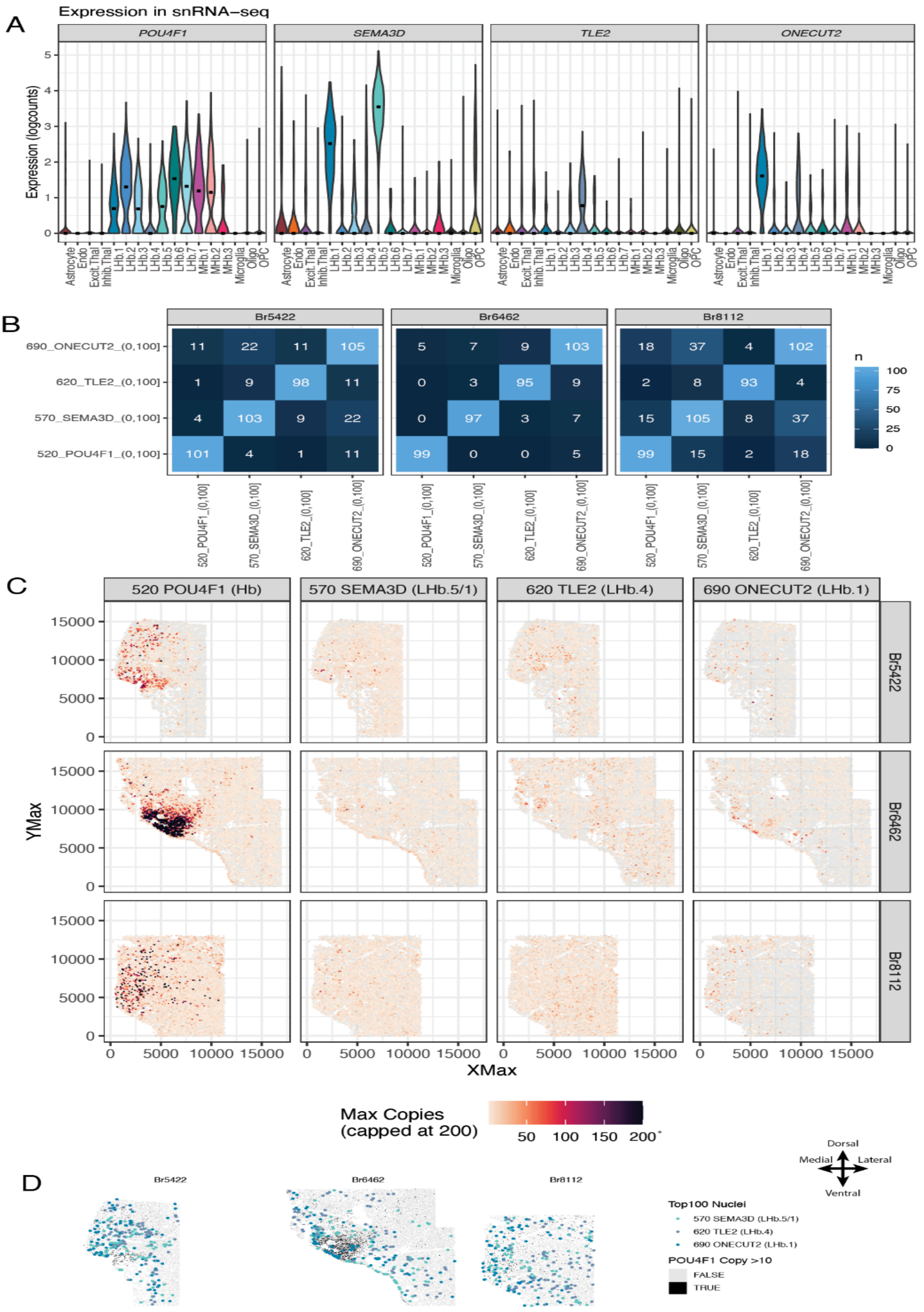

bioRxiv preprint doi: https://doi.org/10.1101/2024.02.26.582081; this version posted February 27, 2024. The copyright holder for this preprint (which was not certified by peer review) is the author/funder, who has granted bioRxiv a license to display the preprint in perpetuity. It is made available under aCC-BY 4.0 International license.

Supplementary Figure 6: Lateral habenula smFISH to visualize LHb.1, LHb.4, and LHb.5 subpopulations. A) Violin plots of selected mean ratio marker gene expression (in normalized (`logcounts`)) for *POU4F1, SEMA3D, TLE2, ONECUT2* across cell types identified from the snRNA-seq data. *POU4F1* broadly marks Hb cell populations (labeled with Opal dye 520), *SEMA3D* marks LHb.1/5 (labeled with Opal dye 570), *TLE2* marks LHb.4 (labeled with Opal dye 620), and *ONECUT2* marks LHb.1 (labeled with Opal dye 690). **B**) For each donor tissue section: Confusion matrix of top 100 cells per marker gene probe channel, ranked by number of transcript copies. Non-diagonal squares (dark blue) show number of cells that are in the top 100 of those two marker genes. Ranks include ties, therefore each square along the diagonal (light blue) can be more than 100. **C**) For each marker gene probe channel: Hexbin plots displaying the maximum number of transcript copies per bin across the tissue section from each donor. To aid visualization, all values > 200 copies are the same color. XMax and YMax values provide spatial locations of the cells defined during cell object segmentation in HALO. **D**) Spatial plots displaying the top cells expressing each marker gene (**Fig 3Ai**), with each detected cell in the tissue section plotted as a gray or black rectangle. Cells in black with > 10 transcript copies of the Hb marker gene *POU4F1* depict the habenula region. Colored circles mark the spatial location of the top 100 ranked cells that most robustly express each marker gene probe. If a cell was ranked in the top 100 for more than one marker gene (see **Supplementary Figure 6B**), it was colored by the gene for which it had the most number of transcript copies.

bioRxiv preprint doi: https://doi.org/10.1101/2024.02.26.582081; this version posted February 27, 2024. The copyright holder for this preprint (which was not certified by peer review) is the author/funder, who has granted bioRxiv a license to display the preprint in perpetuity. It is made available under aCC-BY 4.0 International license.

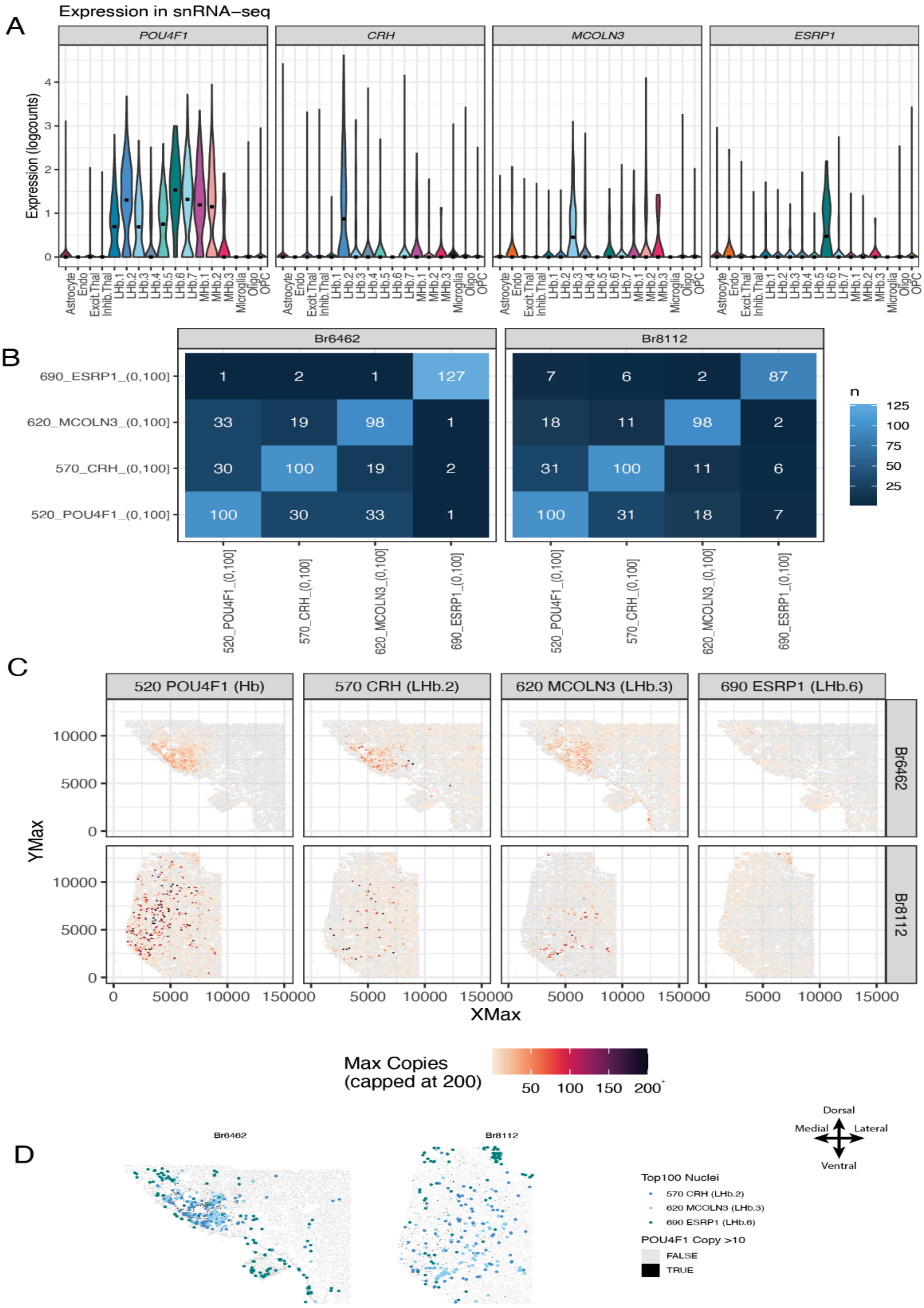

bioRxiv preprint doi: https://doi.org/10.1101/2024.02.26.582081; this version posted February 27, 2024. The copyright holder for this preprint (which was not certified by peer review) is the author/funder, who has granted bioRxiv a license to display the preprint in perpetuity. It is made available under aCC-BY 4.0 International license.

Supplementary Figure 7: Lateral habenula smFISH to visualize LHb.2, LHb.3, and LHb.6 subpopulations. A) Violin plots of selected mean ratio marker gene expression (in normalized (`logcounts`)) for *POU4F1, CRH, MCOLN3, ESRP1* across cell types identified from the snRNA-seq data. *POU4F1* broadly marks Hb cell populations (labeled with Opal dye 520)*, CRH* marks LHb.2 (labeled with Opal dye 570)*, MCOLN3* marks LHb.3 (labeled with Opal dye 620)*,* and *ESRP1* marks LHb.6 (labeled with Opal dye 690). **B)** For each donor tissue section: Confusion matrix of top 100 cells per marker gene probe channel, ranked by number of transcript copies. Non-diagonal squares (dark blue) show number of cells that are in the top 100 of those two marker genes. Ranks include ties, therefore each square along the diagonal (light blue) can be more than 100. **C)** For each marker gene probe channel: Hexbin plots displaying the maximum number of transcript copies per bin across the tissue section from each donor. To aid visualization, all values > 200 copies are the same color. XMax and YMax values provide spatial locations of the cells defined during cell object segmentation in HALO. **D**) Spatial plots displaying the top cells expressing each marker gene (**Fig 3Bi**), with each detected cell in the tissue section plotted as a gray or black rectangle. Cells in black with > 10 transcript copies of the Hb marker gene *POU4F1* depict the habenula region. Colored circles mark the spatial location of the top 100 ranked cells that most robustly express each marker gene probe. If a cell was ranked in the top 100 for more than one marker gene (see **Supplementary Figure 7B**), it was colored by the gene for which it had the most number of transcript copies.

bioRxiv preprint doi: https://doi.org/10.1101/2024.02.26.582081; this version posted February 27, 2024. The copyright holder for this preprint (which was not certified by peer review) is the author/funder, who has granted bioRxiv a license to display the preprint in perpetuity. It is made available under aCC-BY 4.0 International license.

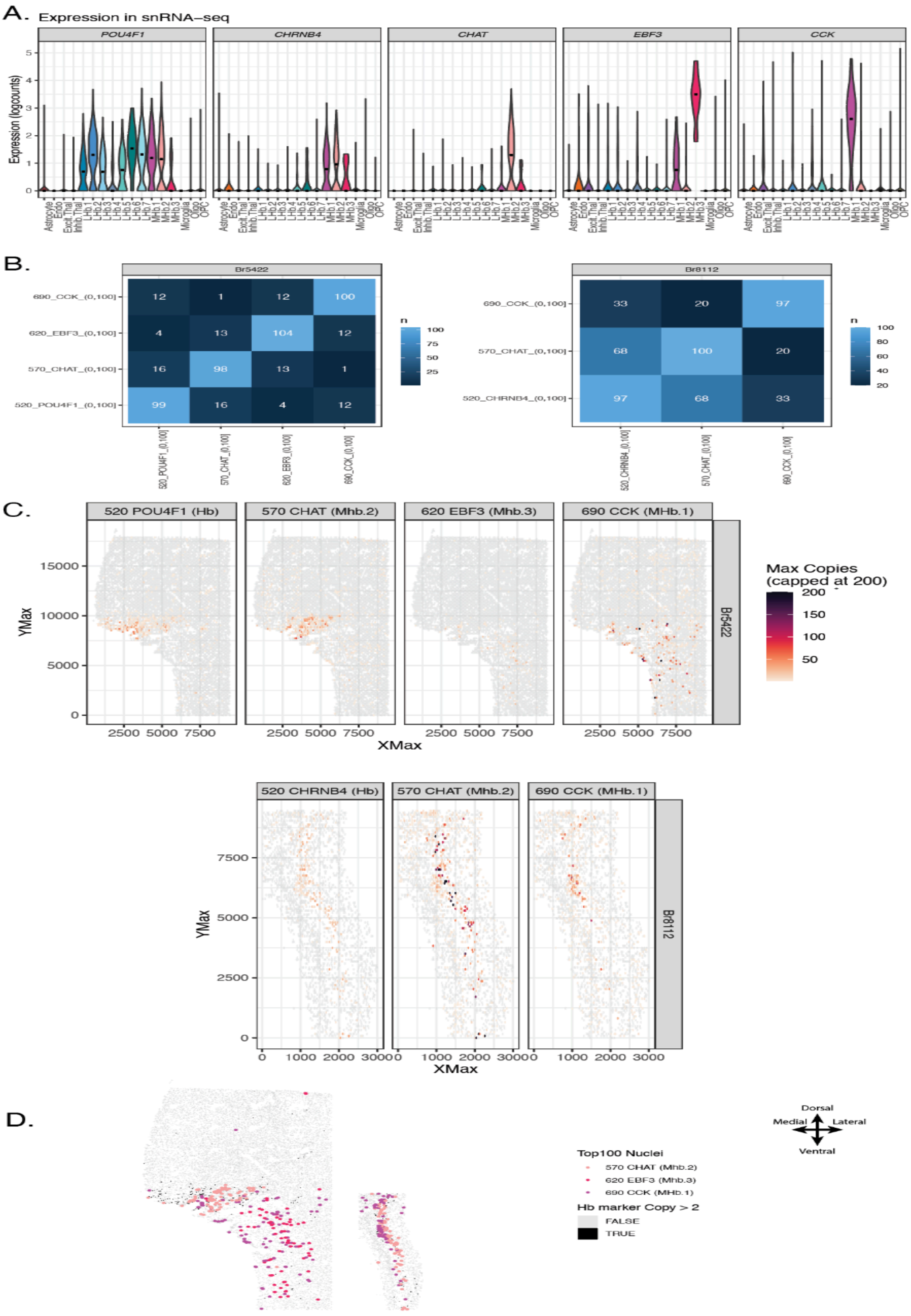

bioRxiv preprint doi: https://doi.org/10.1101/2024.02.26.582081; this version posted February 27, 2024. The copyright holder for this preprint (which was not certified by peer review) is the author/funder, who has granted bioRxiv a license to display the preprint in perpetuity. It is made available under aCC-BY 4.0 International license.

Supplementary Figure 8: Medial habenula smFISH for MHb.1, MHb.2, and MHb.3 subpopulations. A) Violin plots of selected mean ratio marker gene expression (in normalized (`logcounts`)) for *POU4F1, CHAT, EBF3, CCK* across cell types identified from the snRNA-seq data. *POU4F1* broadly marks Hb cell populations (labeled with Opal dye 520)*, CHAT* marks MHb.2 (labeled with Opal dye 570)*, EBF3* marks MHb.3 (labeled with Opal dye 620)*,* and *CCK* marks MHb.1 (labeled with Opal dye 690). **B)** For each donor tissue section: Confusion matrix of top 100 cells per marker gene probe channel, ranked by number of transcript copies. Non-diagonal squares (dark blue) show number of cells that are in the top 100 of those two marker genes. Ranks include ties, therefore each square along the diagonal (light blue) can be more than 100. **C)** For each marker gene probe channel: Hexbin plots displaying the maximum number of transcript copies per bin across the tissue section from each donor. To aid visualization, all values > 200 copies are the same color. XMax and YMax values provide spatial locations of the cells defined during cell object segmentation in HALO. **D**) Spatial plots displaying the top cells expressing each marker gene (**Fig 3Ci**), with each detected cell in the tissue section plotted as a gray or black rectangle. Cells in black with > 2 transcript copies of the Hb marker gene *POU4F1* depict the habenula region. Colored circles mark the spatial location of the top 100 ranked cells that most robustly express each marker gene probe. If a cell was ranked in the top 100 for more than one marker gene (see **Supplementary Figure 8B**), it was colored by the gene for which it had the most number of transcript copies.

bioRxiv preprint doi: https://doi.org/10.1101/2024.02.26.582081; this version posted February 27, 2024. The copyright holder for this preprint (which was not certified by peer review) is the author/funder, who has granted bioRxiv a license to display the preprint in perpetuity. It is made available under aCC-BY 4.0 International license.

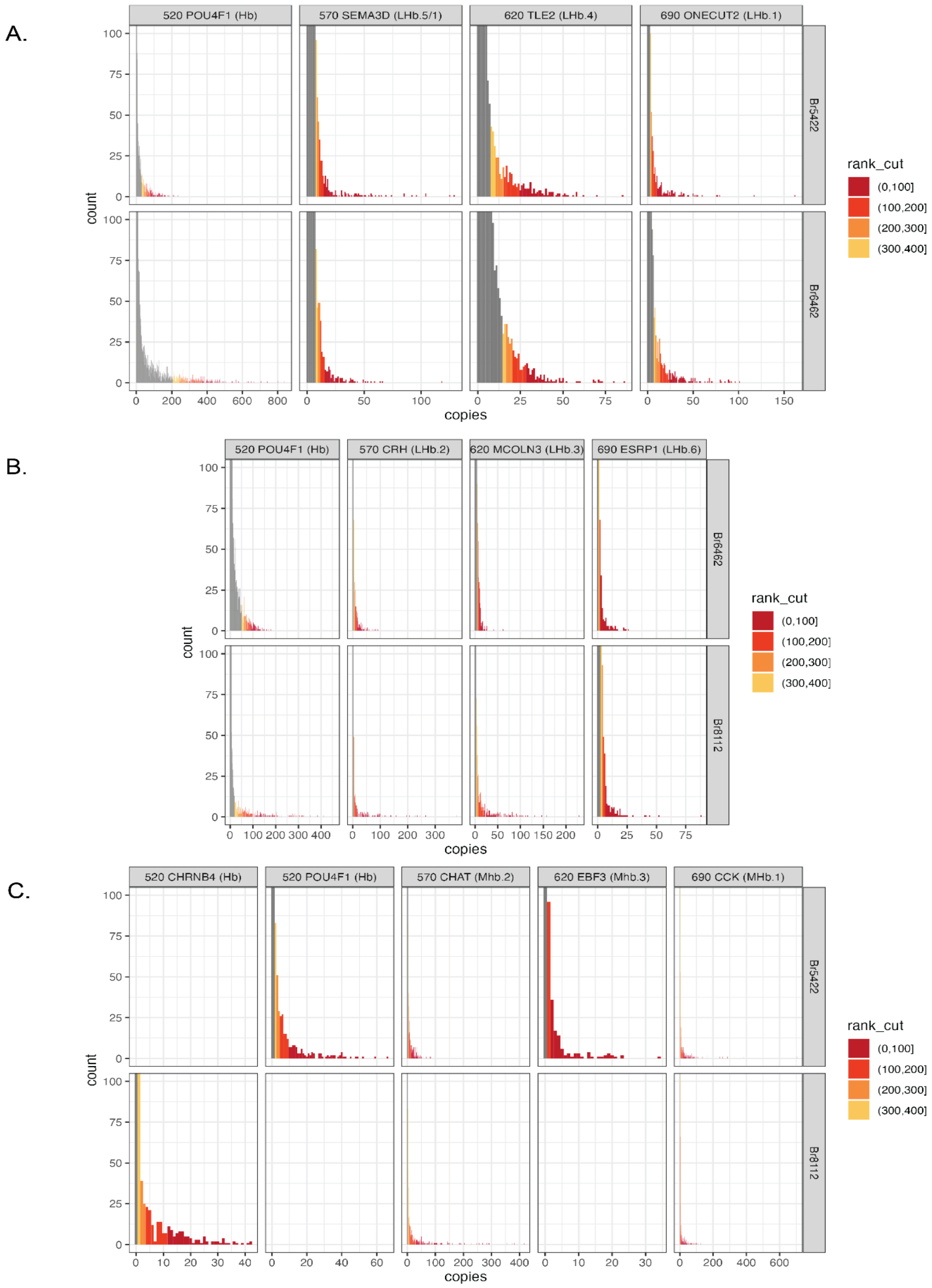

bioRxiv preprint doi: https://doi.org/10.1101/2024.02.26.582081; this version posted February 27, 2024. The copyright holder for this preprint (which was not certified by peer review) is the author/funder, who has granted bioRxiv a license to display the preprint in perpetuity. It is made available under aCC-BY 4.0 International license.

Supplementary Figure 9:: Distributions of marker gene expression estimations across smFISH experiments. HALO software (Indica Labs) was used to detect and quantify RNAScope probes in each cell object. **A)** Frequency count distribution of the estimated number of *POU4F1*, *SEMA3D*, *TLE2*, and *ONECUT2* marker gene copies for each cell object. **B)** Frequency count distribution of the estimated number of *POU4F1*, *CRH*, *MCOLN3*, and *ESRP1* marker gene copies for each cell object. **C)** Frequency count distribution of the estimated number of *POU4F1*, *CHAT*, *EBF3*, *CCK*, and *CHRNB4* marker gene copies for each cell object. In A-C, the count frequencies (y-axis) are limited to 100 in order to observe the frequency counts for non-zero copy values.

bioRxiv preprint doi: https://doi.org/10.1101/2024.02.26.582081; this version posted February 27, 2024. The copyright holder for this preprint (which was not certified by peer review) is the author/funder, who has granted bioRxiv a license to display the preprint in perpetuity. It is made available under aCC-BY 4.0 International license.

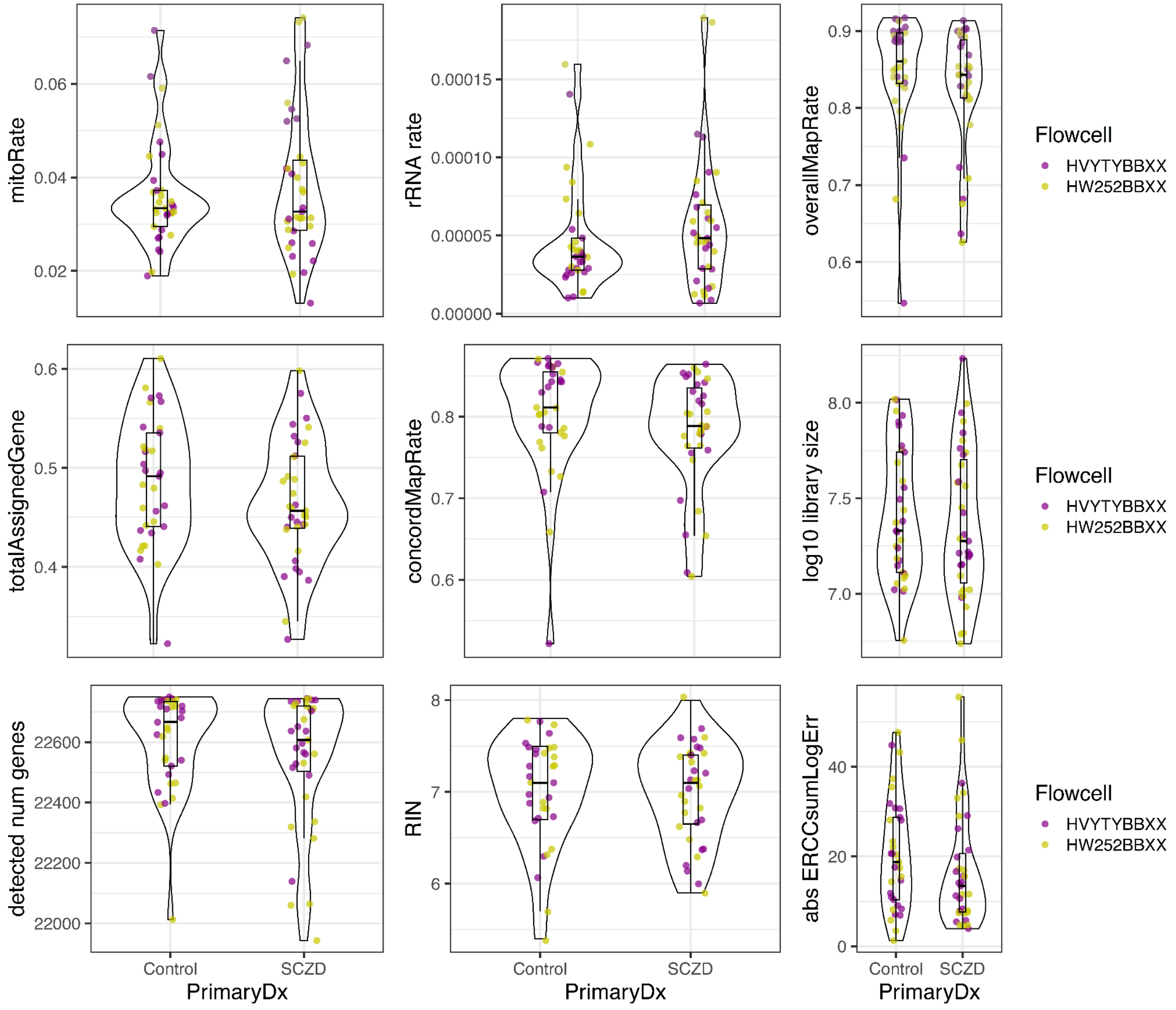

Supplementary Figure 10: Bulk RNA-sequencing quality control metrics. Boxplots and violin distribution graphs for several bulk RNA-seq quality metrics computed by *SPEAQeasy*. Samples are separated by Control vs. SCZD status and colored by the sequencing flowcell. Sample Br5572, which was excluded from analysis, is not included in these graphs.

bioRxiv preprint doi: https://doi.org/10.1101/2024.02.26.582081; this version posted February 27, 2024. The copyright holder for this preprint (which was not certified by peer review) is the author/funder, who has granted bioRxiv a license to display the preprint in perpetuity. It is made available under aCC-BY 4.0 International license.

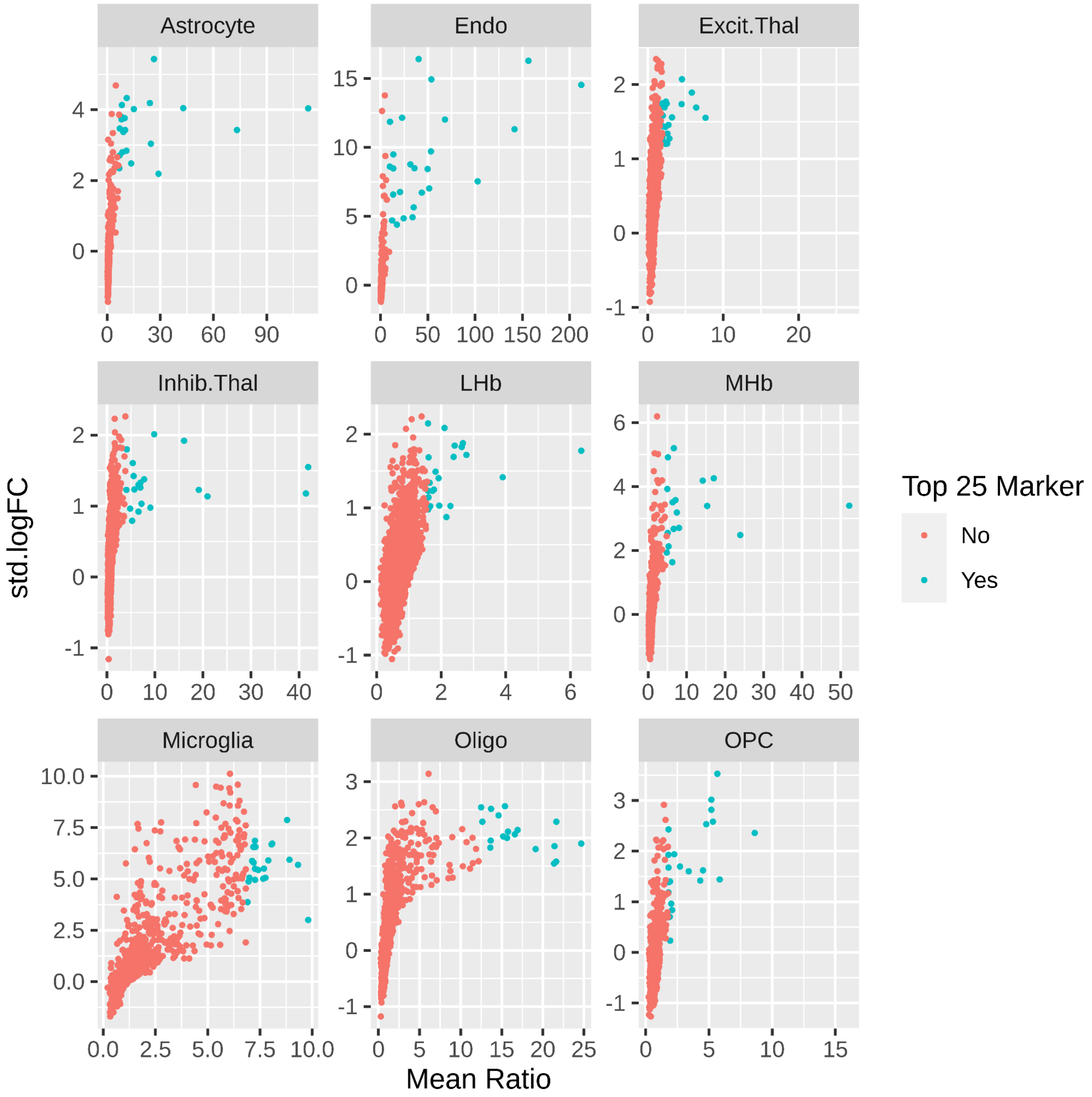

Supplementary Figure 11: Selection of top mean ratio marker genes for cell type deconvolution. For each cell type category, mean ratios for all the detected genes (see **Supplemental Methods**) were computed by `getMeanRatio2()` from *DeconvoBuddies* and compared against the genes' standard log2 fold change values (std.logFC), which were computed by `findMarkers()` from *scran*. The top 25 mean ratio marker genes are shown in teal, and typically have a high standard log2 fold change value.

bioRxiv preprint doi: https://doi.org/10.1101/2024.02.26.582081; this version posted February 27, 2024. The copyright holder for this preprint (which was not certified by peer review) is the author/funder, who has granted bioRxiv a license to display the preprint in perpetuity. It is made available under aCC-BY 4.0 International license.

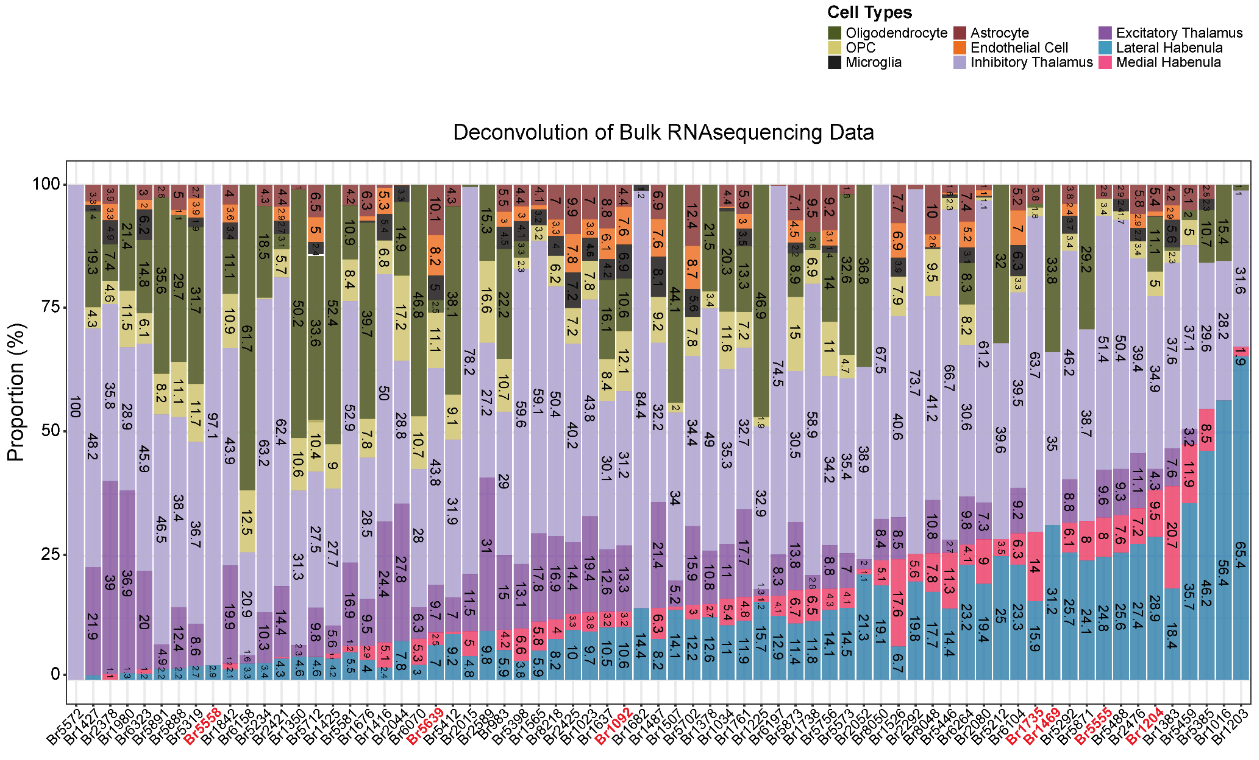

Supplementary Figure 12: Cell type proportions of the bulk RNA-sequencing samples as estimated by deconvolution. Bar plots show the proportions of each cell type in the bulk RNA-seq samples as estimated by deconvolution. Increasing from left to right, samples are ordered by the sum of their estimated LHb (blue) and MHb (pink) proportions. As sample Br5572 was estimated to consist entirely of inhibitory thalamic neurons, we dropped this sample from further analyses. Following this exclusion, we retained 33 Control and 35 SCZD samples. Subjects highlighted in red were also used for snRNA-seq.

bioRxiv preprint doi: https://doi.org/10.1101/2024.02.26.582081; this version posted February 27, 2024. The copyright holder for this preprint (which was not certified by peer review) is the author/funder, who has granted bioRxiv a license to display the preprint in perpetuity. It is made available under aCC-BY 4.0 International license.

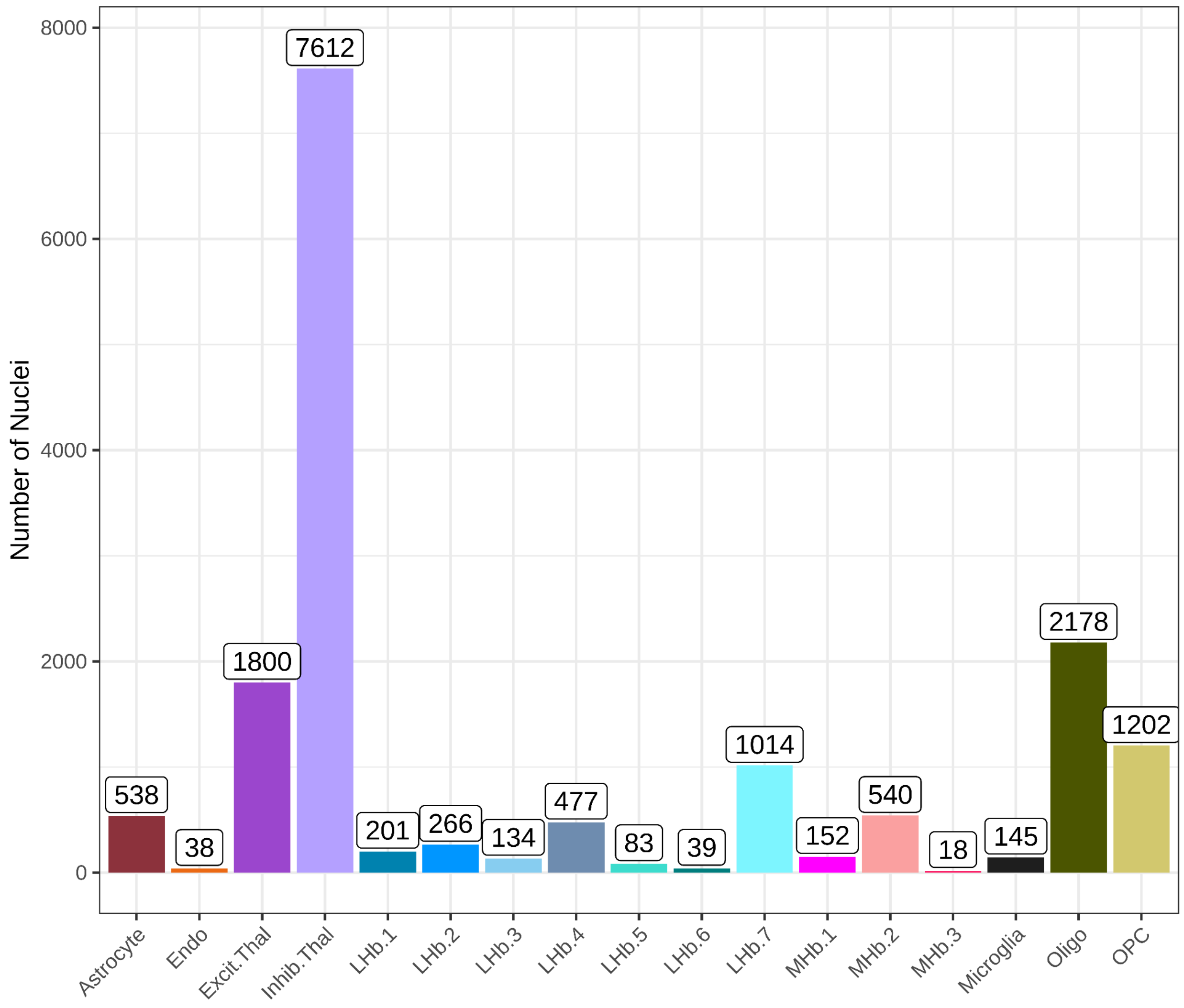

Supplementary Figure 13: snRNA-seq post QC nuclei counts by cell type. Barplots showing the number of nuclei by cell type after quality control steps, aggregated across all seven snRNA-seq samples. Total nuclei post-QC: 16,437.

bioRxiv preprint doi: https://doi.org/10.1101/2024.02.26.582081; this version posted February 27, 2024. The copyright holder for this preprint (which was not certified by peer review) is the author/funder, who has granted bioRxiv a license to display the preprint in perpetuity. It is made available under aCC-BY 4.0 International license.

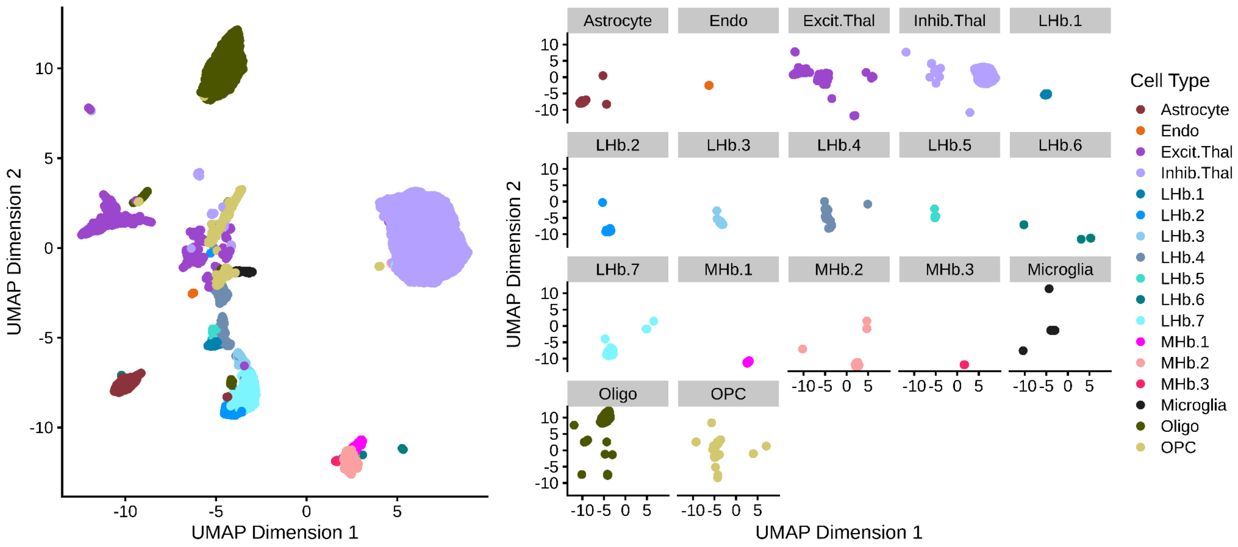

Supplementary Figure 14: UMAP representation of the snRNA-seq data. Post-QC snRNA-seq data visualized using UMAP (similar to **Fig 1**), colored by cell type. The right side shows the UMAP faceted by cell type.

bioRxiv preprint doi: https://doi.org/10.1101/2024.02.26.582081; this version posted February 27, 2024. The copyright holder for this preprint (which was not certified by peer review) is the author/funder, who has granted bioRxiv a license to display the preprint in perpetuity. It is made available under aCC-BY 4.0 International license.

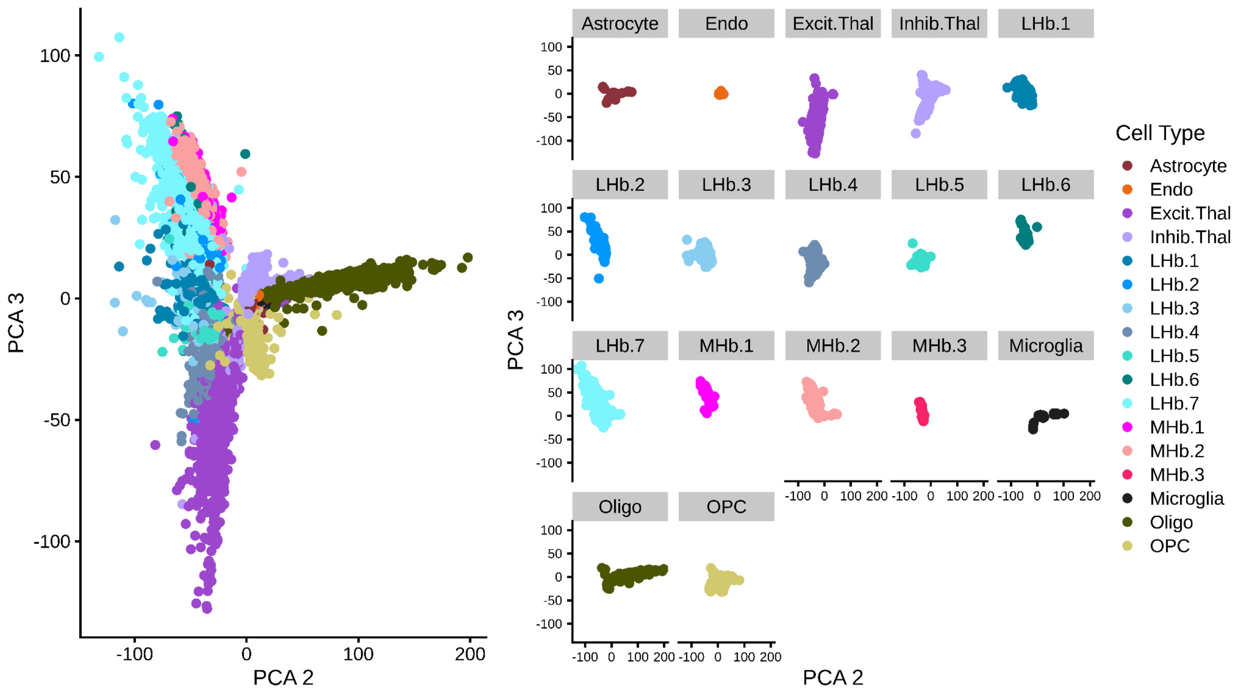

Supplementary Figure 15: snRNA-seq principal components (PCs) by cell type. PC2 versus 3 colored by cell type clusters. The left side of the plot displays all nuclei together, while the right side shows them faceted by cell type. PC2 differentiates non-neuronal cell types (i.e. Oligodendrocytes, Microglia, Astrocytes, Endothelial cells, Oligodendrocyte Progenitor Cells) from neuronal cell types. PC3 differentiates excitatory thalamic neurons from habenula neuron subpopulations.

bioRxiv preprint doi: https://doi.org/10.1101/2024.02.26.582081; this version posted February 27, 2024. The copyright holder for this preprint (which was not certified by peer review) is the author/funder, who has granted bioRxiv a license to display the preprint in perpetuity. It is made available under aCC-BY 4.0 International license.

(A)

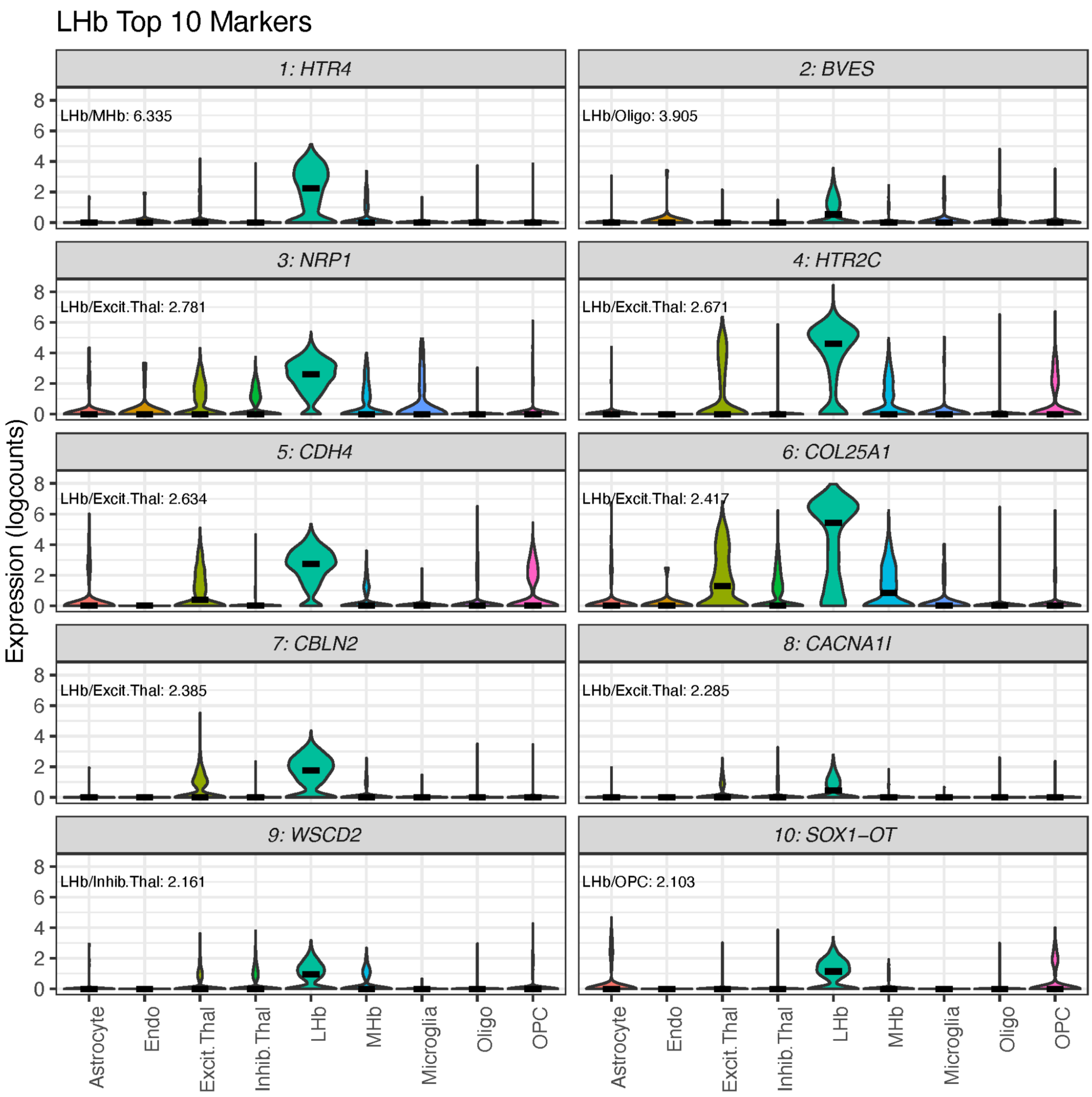

bioRxiv preprint doi: https://doi.org/10.1101/2024.02.26.582081; this version posted February 27, 2024. The copyright holder for this preprint (which was not certified by peer review) is the author/funder, who has granted bioRxiv a license to display the preprint in perpetuity. It is made available under aCC-BY 4.0 International license.

(B)

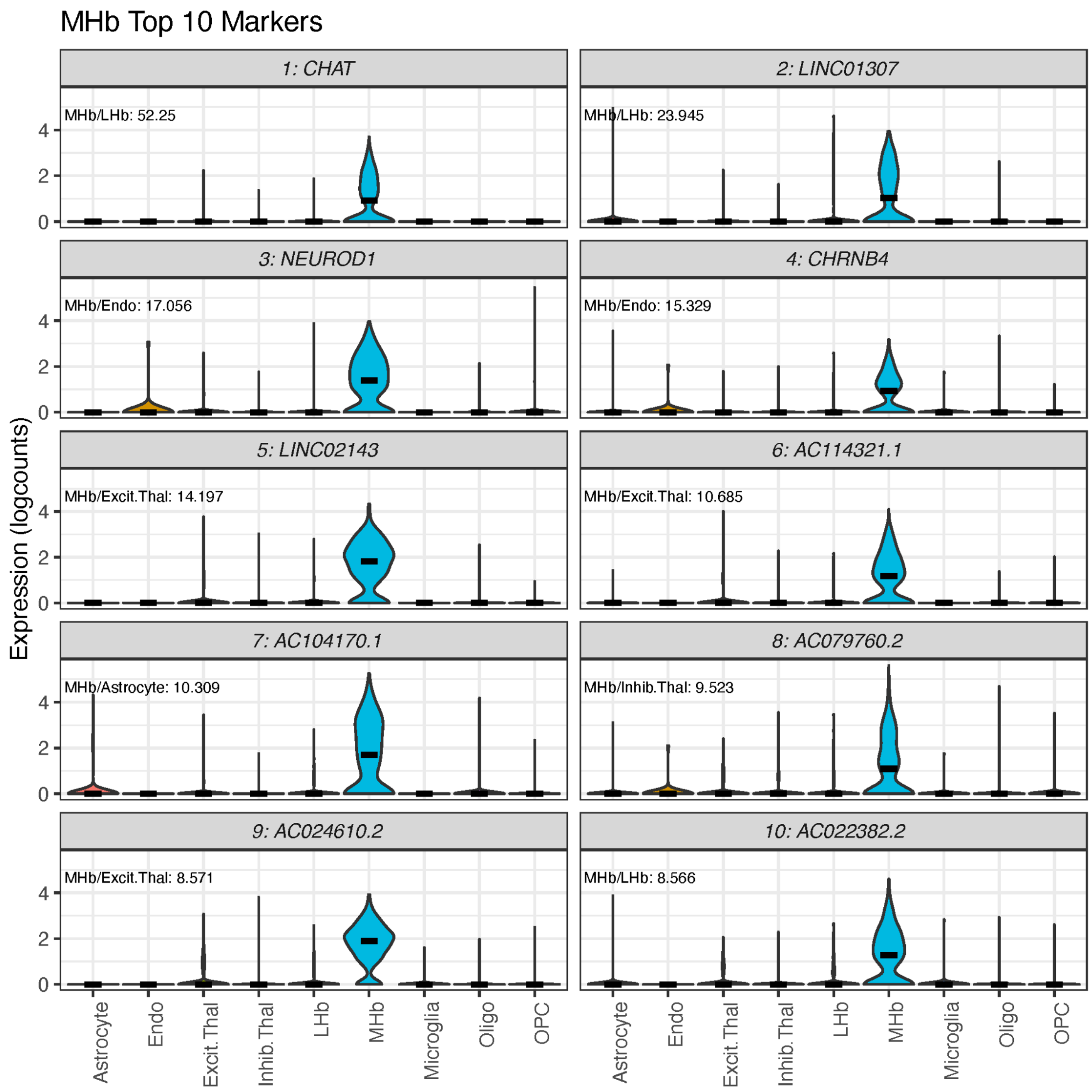

bioRxiv preprint doi: https://doi.org/10.1101/2024.02.26.582081; this version posted February 27, 2024. The copyright holder for this preprint (which was not certified by peer review) is the author/funder, who has granted bioRxiv a license to display the preprint in perpetuity. It is made available under aCC-BY 4.0 International license.

(C)

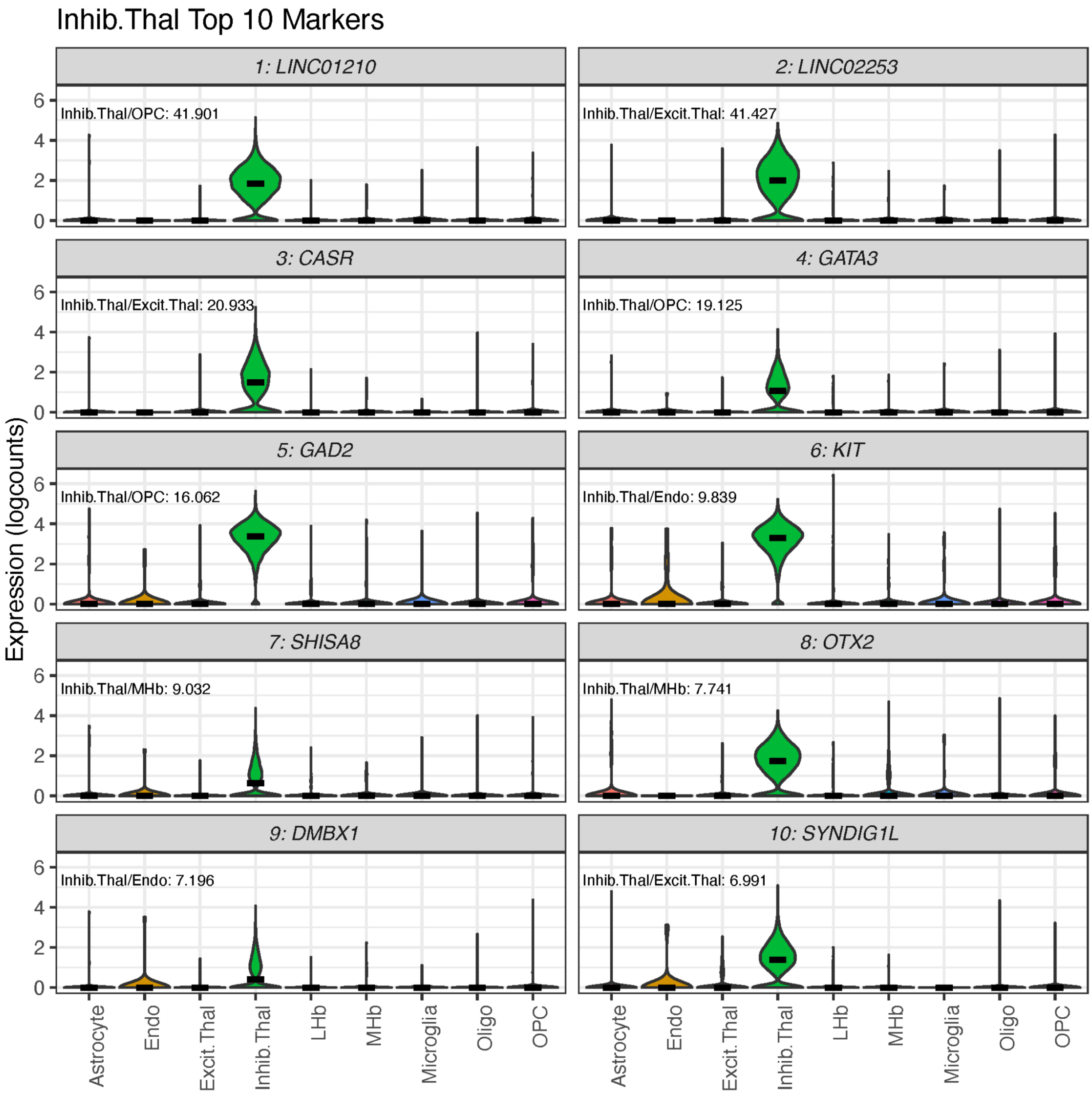

bioRxiv preprint doi: https://doi.org/10.1101/2024.02.26.582081; this version posted February 27, 2024. The copyright holder for this preprint (which was not certified by peer review) is the author/funder, who has granted bioRxiv a license to display the preprint in perpetuity. It is made available under aCC-BY 4.0 International license.

(D)

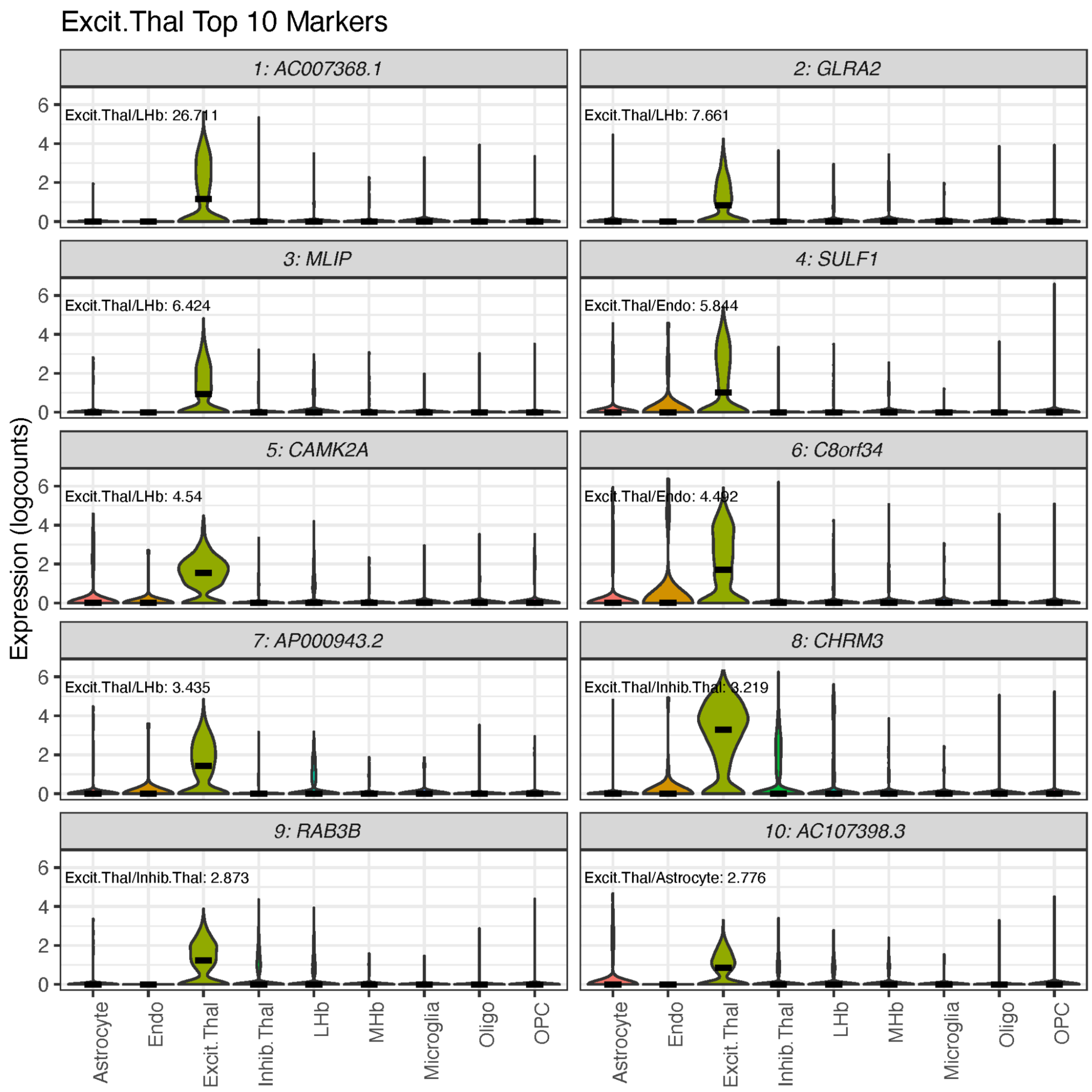

bioRxiv preprint doi: https://doi.org/10.1101/2024.02.26.582081; this version posted February 27, 2024. The copyright holder for this preprint (which was not certified by peer review) is the author/funder, who has granted bioRxiv a license to display the preprint in perpetuity. It is made available under aCC-BY 4.0 International license.

(E)

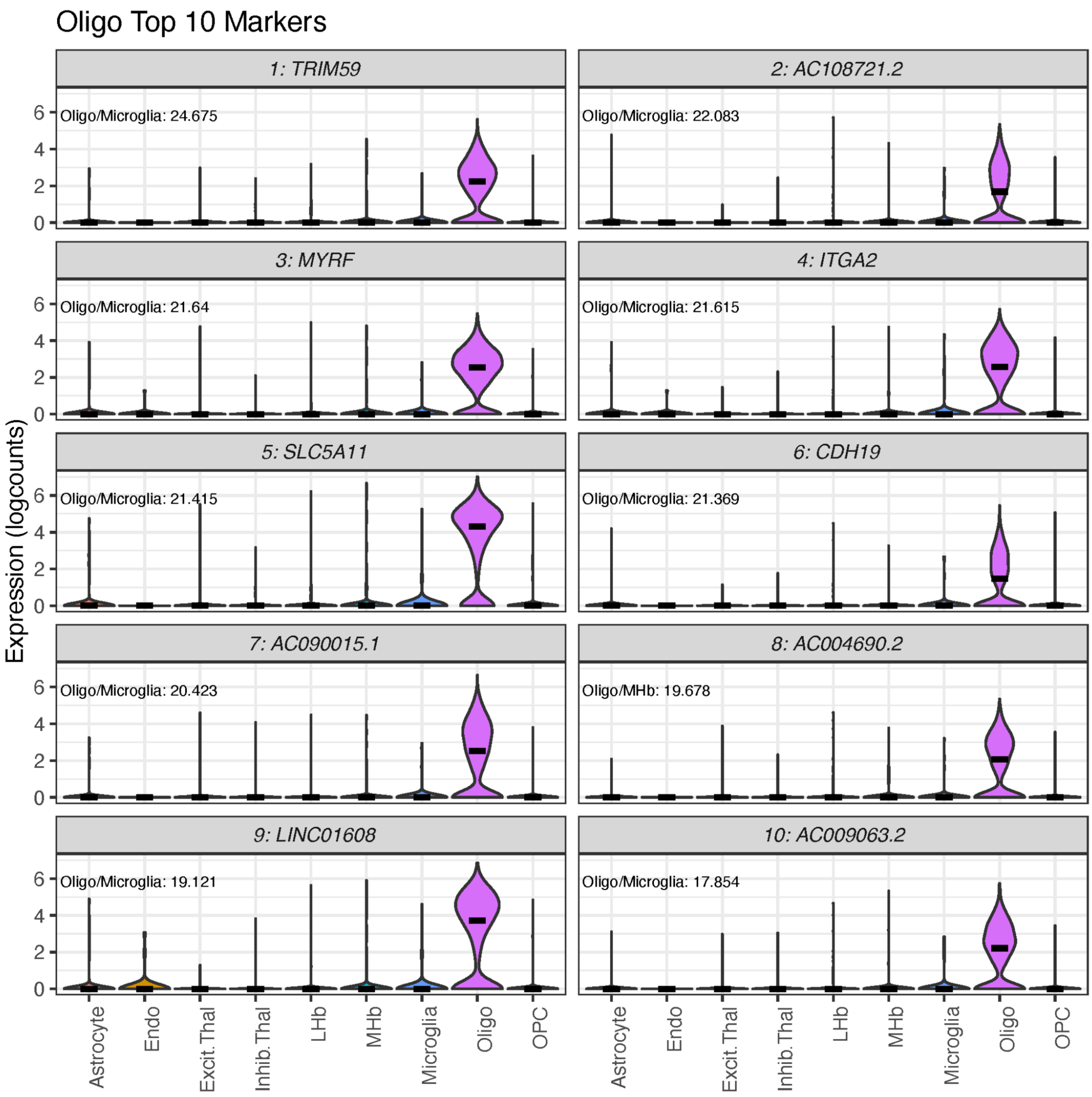

bioRxiv preprint doi: https://doi.org/10.1101/2024.02.26.582081; this version posted February 27, 2024. The copyright holder for this preprint (which was not certified by peer review) is the author/funder, who has granted bioRxiv a license to display the preprint in perpetuity. It is made available under aCC-BY 4.0 International license.

(F)

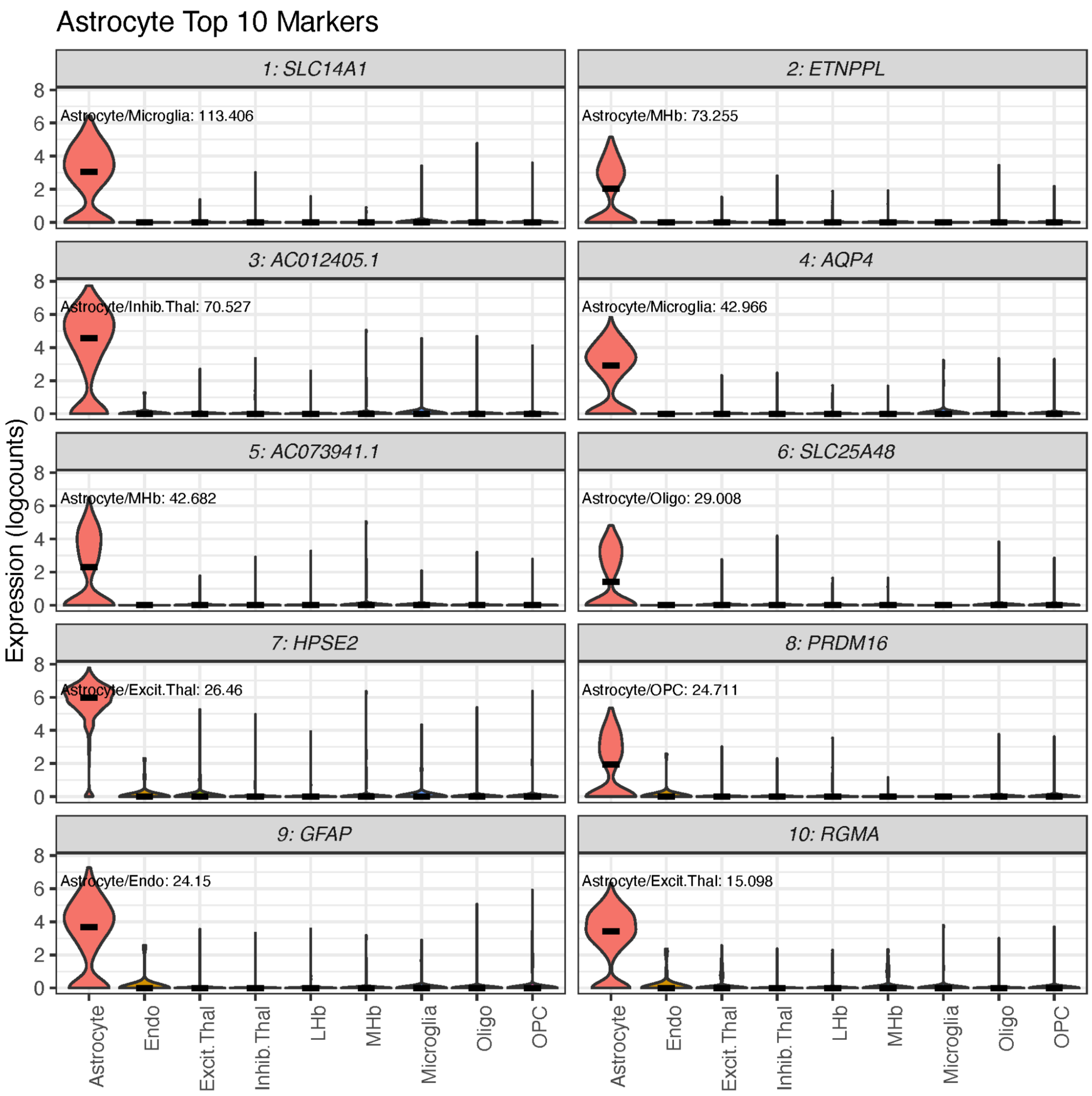

bioRxiv preprint doi: https://doi.org/10.1101/2024.02.26.582081; this version posted February 27, 2024. The copyright holder for this preprint (which was not certified by peer review) is the author/funder, who has granted bioRxiv a license to display the preprint in perpetuity. It is made available under aCC-BY 4.0 International license.

(G)

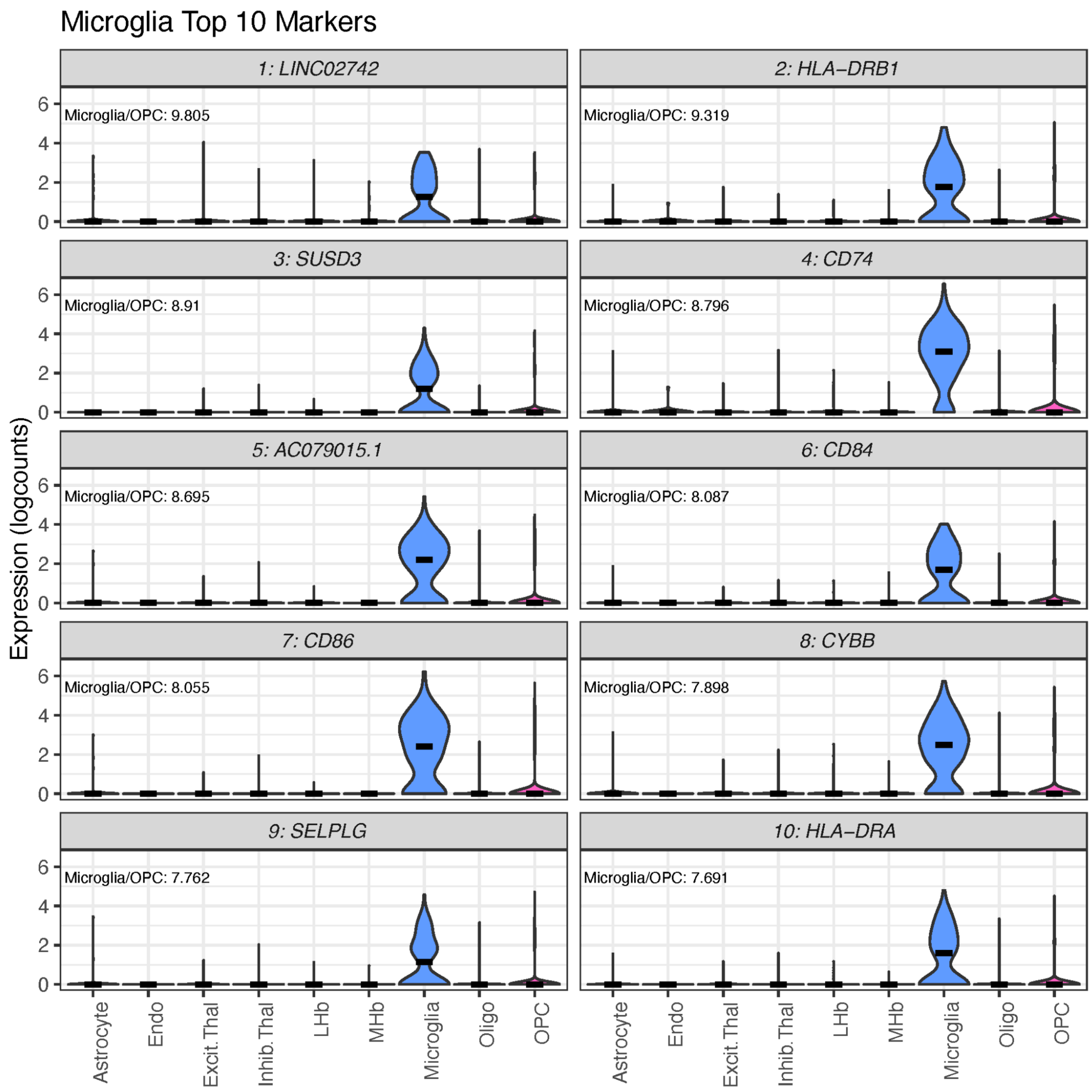

bioRxiv preprint doi: https://doi.org/10.1101/2024.02.26.582081; this version posted February 27, 2024. The copyright holder for this preprint (which was not certified by peer review) is the author/funder, who has granted bioRxiv a license to display the preprint in perpetuity. It is made available under aCC-BY 4.0 International license.

(H)

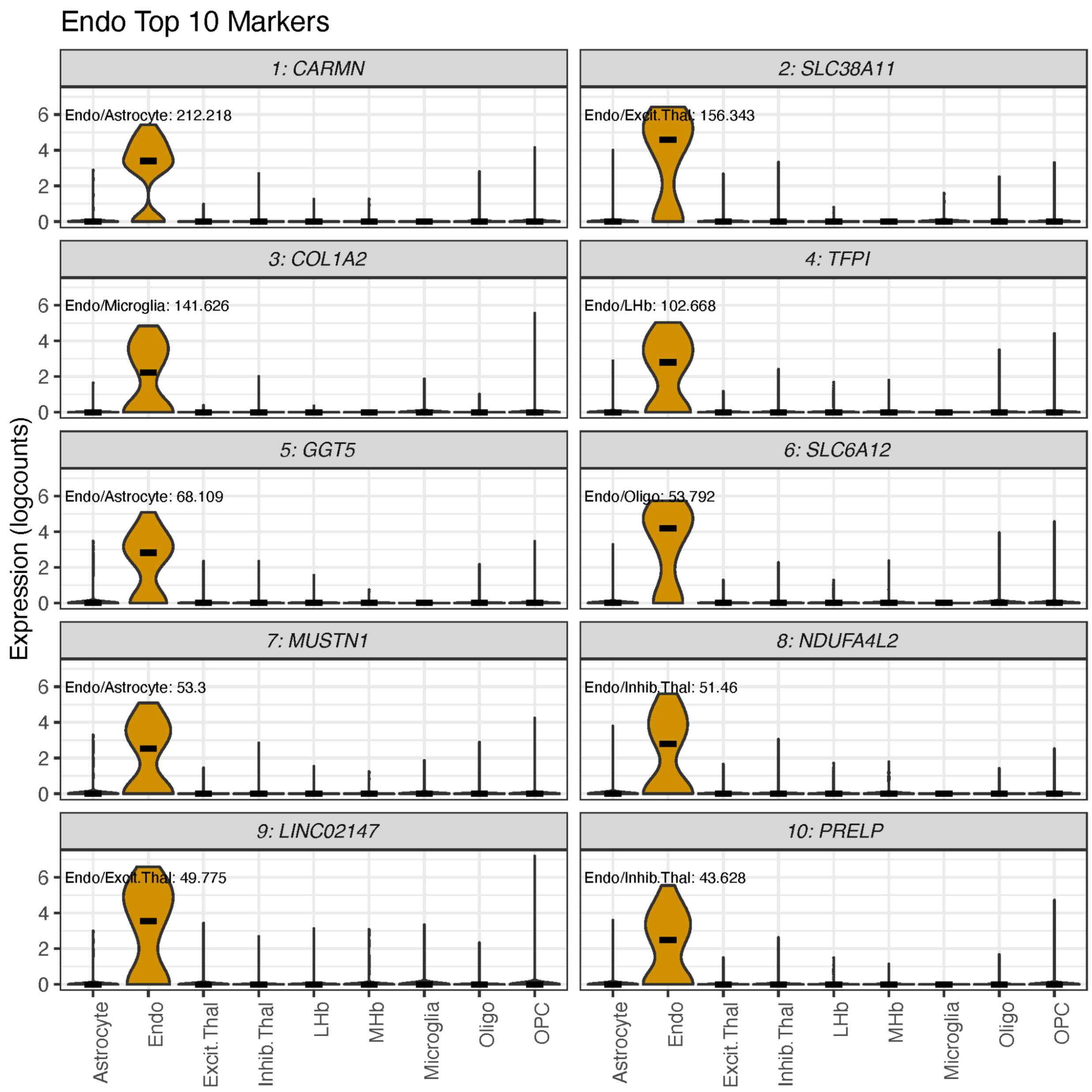

bioRxiv preprint doi: https://doi.org/10.1101/2024.02.26.582081; this version posted February 27, 2024. The copyright holder for this preprint (which was not certified by peer review) is the author/funder, who has granted bioRxiv a license to display the preprint in perpetuity. It is made available under aCC-BY 4.0 International license.

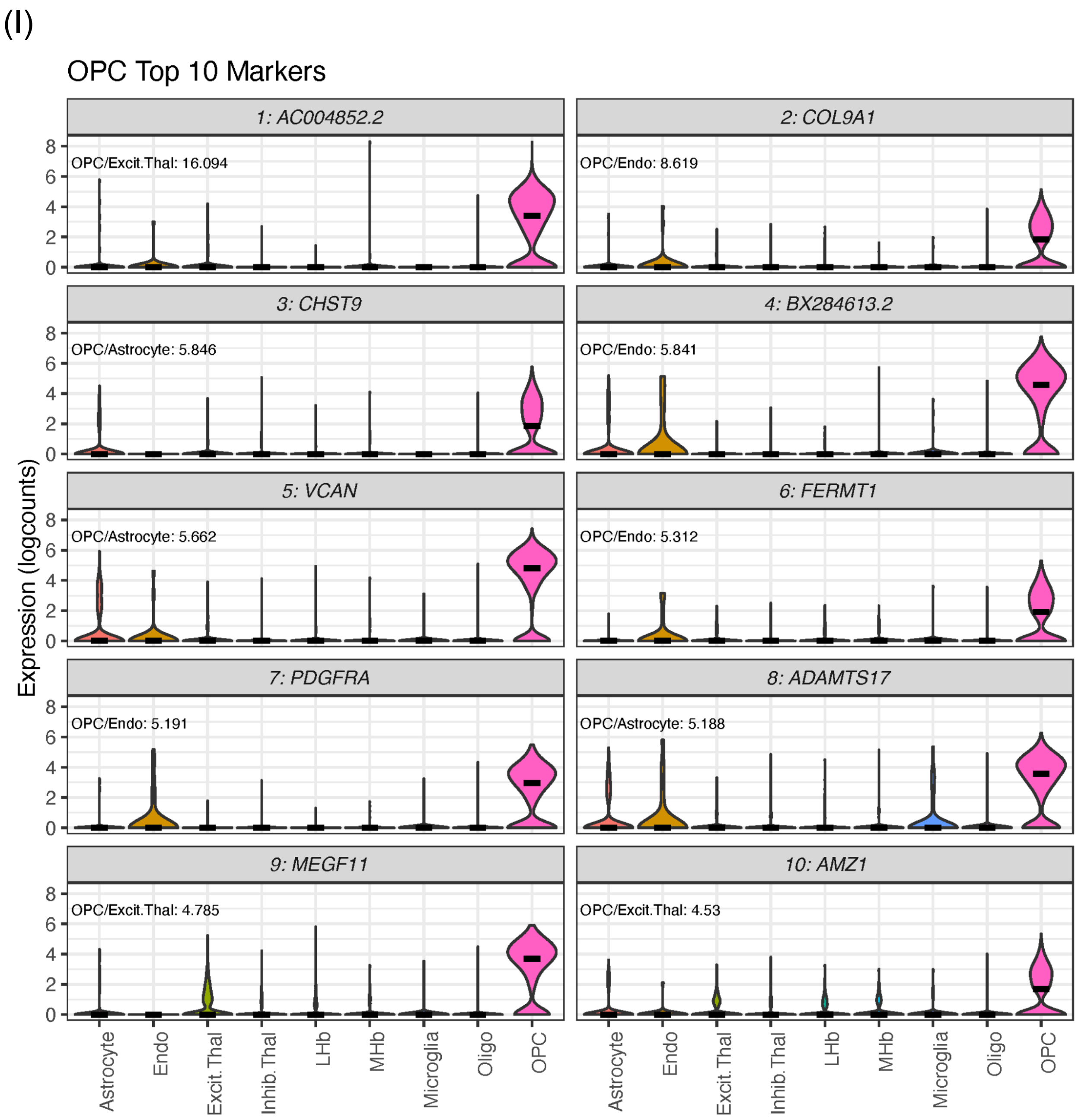

Supplementary Figure 16: Gene expression plots for the top 10 marker genes for each cell type. Violin distribution plots of the log-normalized expression counts (logcounts) of the top 10 mean ratio marker genes for each cell type. Top left corner of each plot shows the target cell type and second-most highly expressing cell type, as well as the ratio of their mean marker gene expression levels (i.e. the mean ratio). **A**) Lateral Habenula neurons (LHb), **B**) Medial Habenula neurons (MHb), **C**) Inhibitory Thalamic neurons (Inhib.Thal), **D**) Excitatory Thalamic neurons (Excit.Thal), **E**) Oligodendrocytes (Oligo), **F**) Astrocytes, **G**) Microglia, **H**) Endothelial cells (Endo), **I**) Oligodendrocyte Progenitor Cells (OPC).

bioRxiv preprint doi: https://doi.org/10.1101/2024.02.26.582081; this version posted February 27, 2024. The copyright holder for this preprint (which was not certified by peer review) is the author/funder, who has granted bioRxiv a license to display the preprint in perpetuity. It is made available under aCC-BY 4.0 International license.

A)

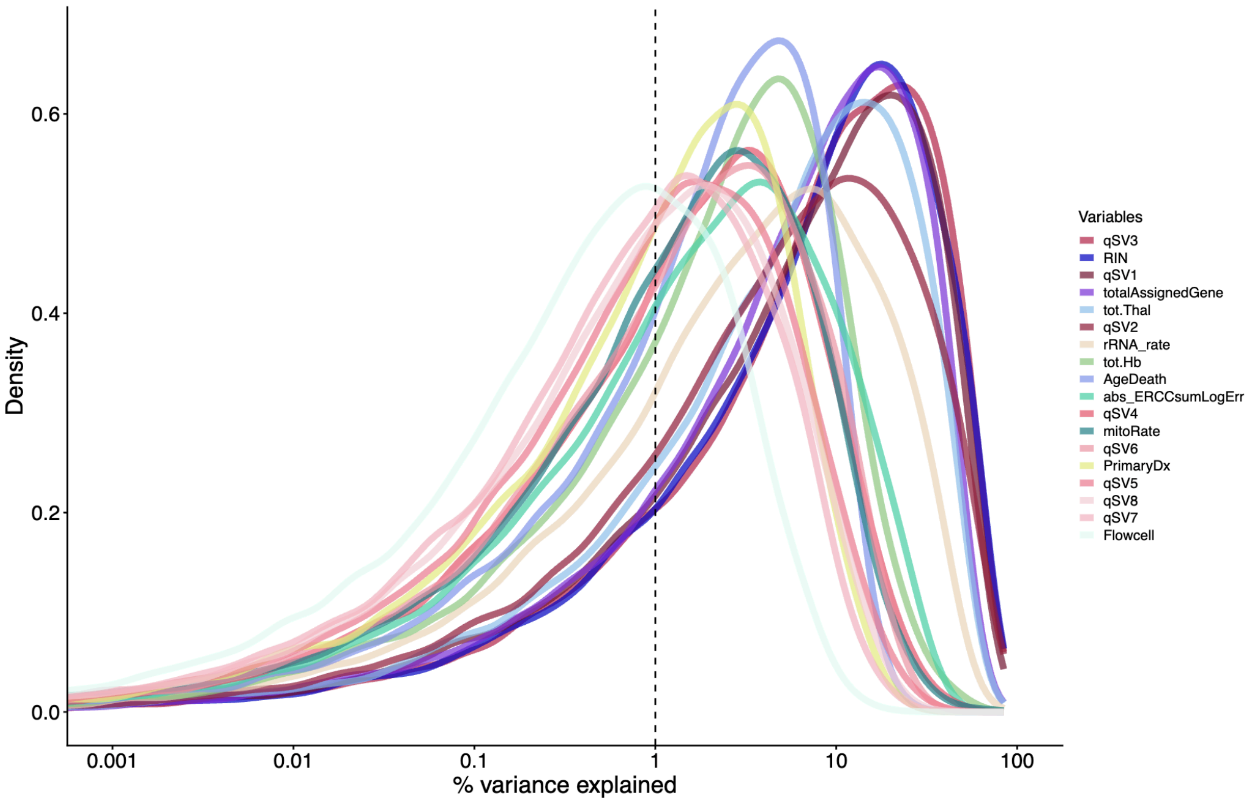

B)

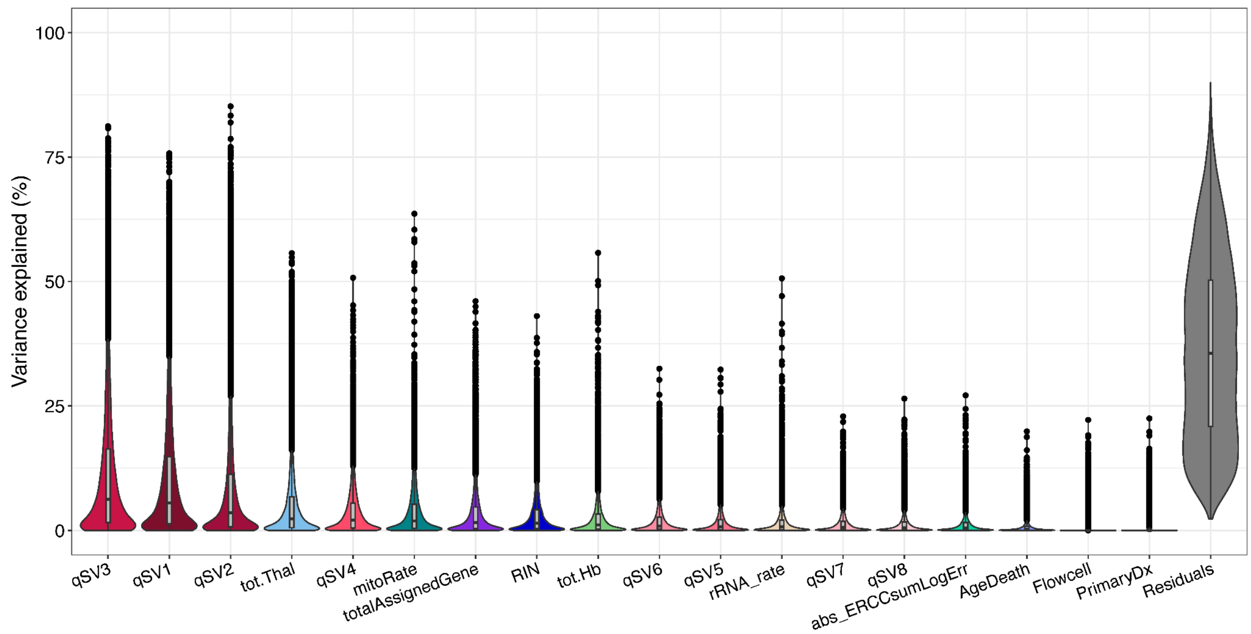

Supplementary Figure 17: Bulk RNA-sequencing variance partition across covariates. A) Density lines for the percent of variance explained by each covariate across all genes, as estimated using `plotExplanatoryVariables()` from the *scater* package. The x-axis is

bioRxiv preprint doi: https://doi.org/10.1101/2024.02.26.582081; this version posted February 27, 2024. The copyright holder for this preprint (which was not certified by peer review) is the author/funder, who has granted bioRxiv a license to display the preprint in perpetuity. It is made available under aCC-BY 4.0 International license.

displayed on a $\log_{10}$ scale. The RNA Integrity Number (RIN), quality Surrogate Variables (qSV) 1 and 3, percent of reads assigned to genes (totalAssignedGene), and total percent of Thalamus cell types estimated by deconvolution (tot.Thal) are among the covariates that explain the highest percent of variance. **B**) Variance explained distribution boxplots across all genes for each of the covariates, as estimated with the *variancePartition* package.

bioRxiv preprint doi: https://doi.org/10.1101/2024.02.26.582081; this version posted February 27, 2024. The copyright holder for this preprint (which was not certified by peer review) is the author/funder, who has granted bioRxiv a license to display the preprint in perpetuity. It is made available under aCC-BY 4.0 International license.

Supplementary Tables

ETable 1: **Donor demographics and bulk RNA-sequencing *SPEAQeasy* metrics.** Donor ID (BrNum), bulk RNA-seq library ID (RNum), age at time of death, sex, race, and primary diagnosis (SCZD vs. Control) are the demographics included. *SPEAQeasy* metrics are documented at https://research.libd.org/SPEAQeasy/outputs.html#quality-metrics. The bulk RNA-seq sequencing flowcell, metrics derived from the bulk RNA-seq QC analysis with *scran,* deconvolution results with *BisqueRNA*, quality surrogate variables (qSVs) obtained with *qsvaR*, and the first 10 DNA genotype ancestry principal components (snpPCs) are also included. All snRNA-seq samples are a subset of these donors.

ETable 2: CellRanger snRNA-seq metrics. Metrics computed by *CellRanger* v6.0.0 for the seven snRNA-seq samples. For more details about these metrics see: https://www.10xgenomics.com/support/software/cell-ranger/latest/analysis/outputs/cr-3p-outputs-metrics-count

ETable 3: Top 50 mean ratio marker genes by fine cell type category. For each of the seventeen cell type categories – including the finer habenula cell type subclusters – the top 50 mean ratio marker genes are shown.

ETable 4: Donor demographics for RNAScope smFISH experiments. Brain donor ID (BrNum), age at time of death (in years), sex, race, primary diagnosis, estimated postmortem interval (PMI, in hours), and RNA integrity number (RIN) screened on the Prefrontal Cortex (PFC) are provided for the three independent donors used in these experiments.

ETable 5: RNAScope Experiments Summary. Experimental design for RNAScope experiments. Probes, catalog numbers, channel and Opal dye assignments, and dye concentrations used for each probe combination.

ETable 6: Top 50 mean ratio marker genes by broad cell type category. For each of the nine broad cell type categories, the top 50 mean ratio marker genes are shown. Only the top 25 mean ratio marker genes by cell type were used for estimating the cell type proportions by deconvolution.

ETable 7: Bulk RNA-seq schizophrenia vs. control differential gene expression (DGE) analysis results. Harmonized gene-level results for the Habenula (Hb), dorsolateral Prefrontal Cortex (DLPFC) and Hippocampus (HIPPO) from BrainSEQ Phase II, Caudate from BrainSEQ Phase III, and Dentate Gyrus (DG). This analysis was restricted to genes expressed in the Hb.
